# Inhibition of Cell Motility by Cell-Penetrating Dynamic Covalent Cascade Exchangers: Integrins Participate in Thiol-Mediated Uptake

**DOI:** 10.1021/jacsau.3c00113

**Published:** 2023-04-12

**Authors:** Filipe Coelho, Saidbakhrom Saidjalolov, Dimitri Moreau, Oliver Thorn-Seshold, Stefan Matile

**Affiliations:** † Department of Organic Chemistry, University of Geneva, 1211 Geneva, Switzerland; ‡ Department of Biochemistry, University of Geneva, 1211 Geneva, Switzerland; § Department of Pharmacy, Ludwig-Maximilians University of Munich, 81377 Munich, Germany

**Keywords:** cell motility, thiol-mediated uptake, integrins, inhibitors, dynamic covalent inhibitors

## Abstract

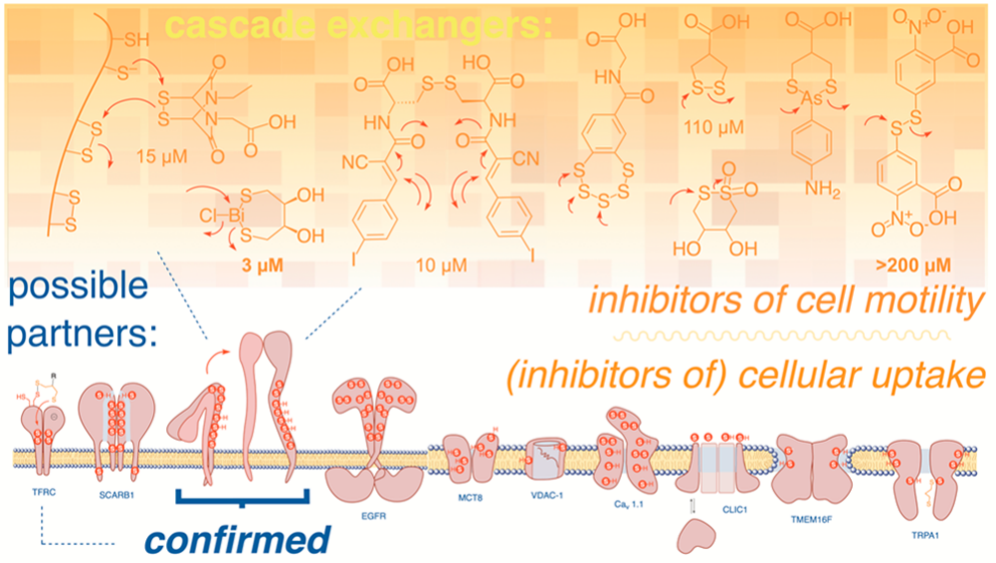

Integrins are cell surface proteins responsible for cell motility. Inspired by the rich disulfide exchange chemistry of integrins, we show here the inhibition of cell migration by cascade exchangers (CAXs), which also enable and inhibit cell penetration by thiol-mediated uptake. Fast-moving CAXs such as reversible Michael acceptor dimers, dithiabismepanes, and bioinspired epidithiodiketopiperazines are best, much better than Ellman’s reagent. The implication that integrins participate in thiol-mediated uptake is confirmed by reduced uptake in integrin-knockdown cells. Although thiol-mediated uptake is increasingly emerging as a unifying pathway to bring matter into cells, its molecular basis is essentially unknown. These results identify the integrin superfamily as experimentally validated general cellular partners in the dynamic covalent exchange cascades that are likely to account for thiol-mediated uptake. The patterns identified testify to the complexity of the dynamic covalent networks involved. This work also provides chemistry tools to explore cell motility and expands the drug discovery potential of CAXs from antiviral toward antithrombotic and antitumor perspectives.

Integrins are cell surface proteins in charge of cell adhesion, cell motility, and bidirectional signaling.^1,2^ Because of their involvement in wound healing, thrombosis,^3^ and cancer cell migration^4^ as well as viral entry^5−10^ and drug delivery,^11^ they are of great interest as drug targets.^2^ In vertebrates, the integrin superfamily consists of 24 heterodimers, all composed of α and β subunits (Figure 1A). They interact with different components of the extracellular matrix. During cell adhesion and migration, integrins change from a bent to a linear conformation. This change is controlled by one of the most beautiful disulfide tracks in biology, composed of 20 disulfides in the β subunit (Figure 1B).^12−15^ Several of these disulfides are allosteric in nature and in a hook or staple conformation,^3,16^ and literature describing dithiol/disulfide redox regulation of integrin function is increasingly emerging.^4,17−20^

**Figure 1:**
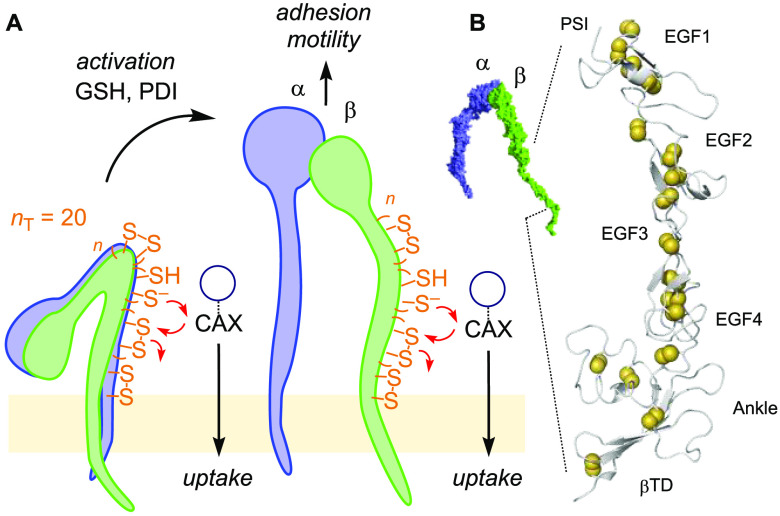
(A) Schematic structure of integrins in inactive (left) and active form (right), highlighting thiol-mediated activation of motility and our hypothesis of TMU by CAX moving along integrin disulfide tracks. (B) Integrins modeled from Protein Data Bank data (PDB entries 3fcs, 2vdo, 2k9j, 2h7d), zoomed onto the disulfide track and indicating domains (yellow, disulfides; TD, tail domain; EGF, epithelial growth factor; PSI, plexin–semaphorin–integrin).^12−15^

Admiring their disulfide tracks, we thought that integrins would be perfect exchange partners in thiol-mediated uptake (TMU). TMU^21−25^ refers to the cell-penetrating activity provided by thiol/disulfide cascade exchanger (CAX) motifs. CAXs undergo dynamic covalent exchanges with membrane-bound protein thiols (or disulfides), where each exchange produces a new (or offers another) covalently tethered exchanger, that can continue exchanging until they are delivered into the cytosol.^21^ TMU has been realized with many classes of CAX for the cytosolic delivery of small molecules,^21,26^ antibodies^22^ and other proteins,^27−30^ genome editing machinery^31^ and other oligonucleotides,^32−37^ polymers,^38^ liposomes,^39^ and nanoparticles^22,40,41^ into various cellular targets including deep tissue,^30,33^ living animals,^31,42^ plant cells,^35^ and bacteria.^26^ Proteomics data,^43^ heatmap patterns,^44,45^ and literature on oligonucleotide phosphorothioate^37,46^ and viral uptake^8,47−50^ all support that multipartner exchange networks are involved in how TMU brings matter into cells.^21^ However, the dynamic covalent exchange cascades of TMU are complex and its fleeting intermediates are elusive, which is likely the reason why TMU is not better known and understood.^21^

Importantly, CAX-induced TMU can be inhibited by treating cells with surface-thiol-reactive agents.^51,52^ Given that thiol-rich integrins are natively poised for reversible thiol modifications,^3,4,16−20^ we hypothesized that CAXs which enable or inhibit TMU could also inhibit the cell motility for which integrin dynamics are crucial, which in turn would demonstrate the involvement of integrins as one of the so far essentially unknown cellular exchange partners in TMU.

To test the effects of TMU inhibitors on cellular integrin functions, compounds **1**–**16** were bought or synthesized following reported procedures (Figures 2 and S2).^44,45,51−55^ Cell motility assays^4^ were adapted to automated high-content high-throughput (AHCHT) imaging methods.^45,51^ Before cell seeding, 96-well plates were coated with one of three proteins that interact with different integrins: Collagen I (C), which activates mainly β_1_-containing integrins; fibronectin (F), which activates mainly β_1_- and β_3_-containing integrins; and vitronectin (V), which activates mainly β_3_- and β_5_-containing integrins.^1−4^ HeLa Kyoto (HK), MCF-7, and MDA-MB-231 cells were then seeded on the protein-coated wells to test their migration behavior when treated with TMU inhibitors. Among many possibilities, highly aggressive MDA-MB-231 and noninvasive MCF-7 breast cancer cells were selected as established standards to explore motility^4^ and HK cells as a link to uptake^45^ and to probe the power of AHCHT assays to detect small changes accurately.

**Figure 2:**
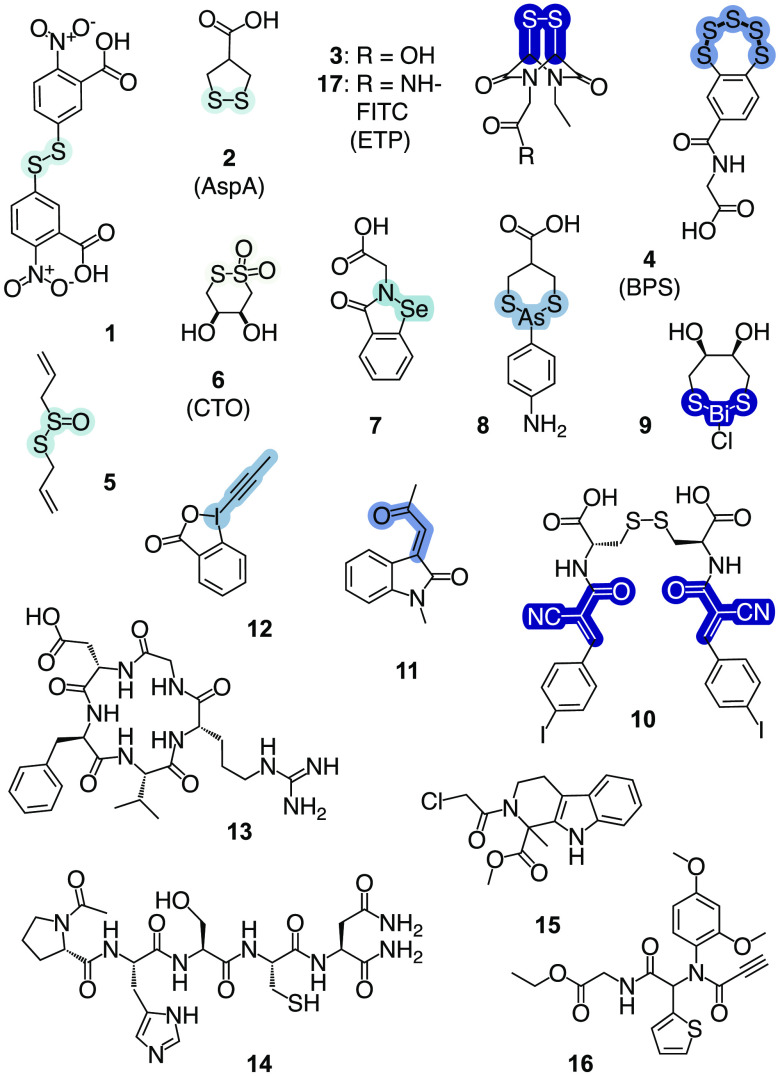
Dynamic covalent inhibitor candidates **1**–**11** and irreversible **12** ordered by relays (**1**–**7**, chalcogens; **8** and **9**, pnictogens; **10**–**12**, tetrels) and activity (increasing left to right, darkening blue; Figure 3G), above established integrin (**13**, **14**) and PDI inhibitors (**15**, **16**). Full structure of TMU reporter **17**: Figure S2.

The confluent cell monolayers were scraped to create “scratches” with widths of around 1 mm using a homemade device that removes cells without damaging the coating (Figure S1), as demonstrated by the different intrinsic motilities of different cells on different coatings (e.g., Figure S14). Cells were then incubated with TMU inhibitor candidates from *t* = 0, and the cell motility *m* was determined from the change of cell-free area *A* from that at *t* = 0 (*m* = *A*_0_ – *A*_*t*_) (Figures 3A,B and S8–S28).

**Figure 3:**
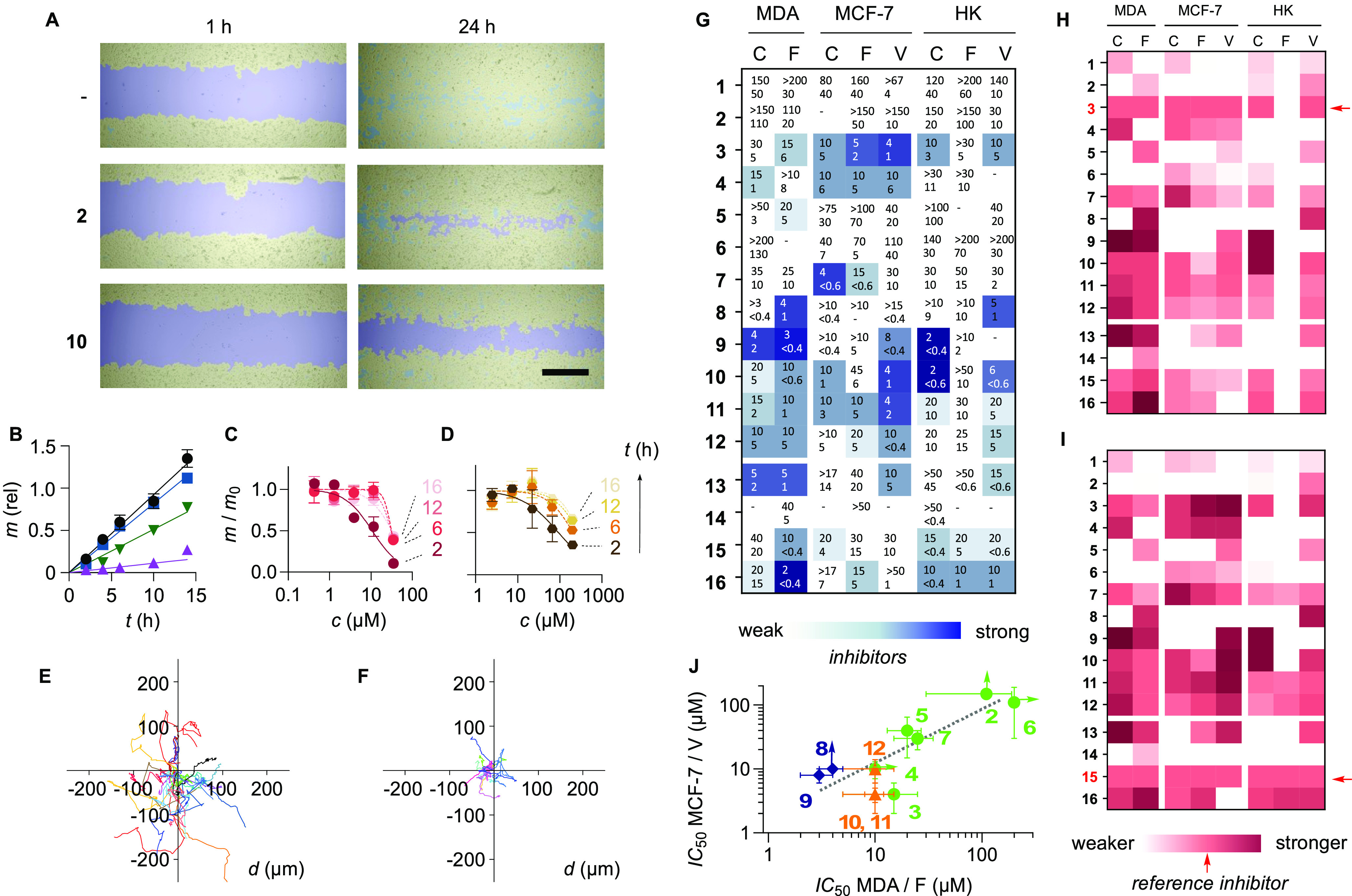
(A) Original transmitted light images for MDA-MB-231 cells on collagen I-coated surfaces without (top) and with inhibitors **2** (150 μM) and **10** (5 μM) 1 and 24 h after scratching, overlaid with automatically generated image masks for cells (yellow) and cell-free area originating from scratch (area = *A*, blue) or interstitial space (cyan). Scale bar: 1 mm. (B) Motility *m* of MDA-MB-231 cells on collagen with **2** (blue squares, 150 μM), **7** (green downward triangles, 50 μM), or **10** (purple upward triangles, 50 μM) or without inhibitor (black circles, =*m*_0_). (C, D) Relative motility *m*/*m*_0_ on collagen for (C) HK cells with **3** and (D) MCF-7 cells with **6** as a function of concentration and time. (E, F) Wind-rose plots with tracks of single MDA-MB-231 cells on collagen I in the (E) absence and (F) presence of **9** (3 μM). (G) Heatmap for the inhibition of the motility of MDA-MB-231, MCF-7, and HK cells on collagen I [C], fibronectin [F], and vitronectin [V] by inhibitor candidates **2**–**12** and controls (**1**, **13**–**16**), measured 2 h after addition at varied concentrations to determine IC_50_ (top) and MIC (=IC_15_, bottom), both in μM. “–”: inactive. (H) Fractional heatmap against **3**. (I) Fractional heatmap against **15**, arrows indicate the reference inhibitors. (J) Two-component correlation of MDA-MB-231 cells on fibronectin against MCF-7 on vitronectin (green circles, chalcogens; blue diamonds, pnictogens; orange triangles, tetrels; error bars, SEM; upward and rightward arrows indicate the actual values to be much higher).

A complex segmentation pipeline was developed to generate unbiased image masks to estimate *A*_t_ (Figures S4–S6). This AHCHT motility assay was particularly powerful with slower moving cells that are otherwise not easily distinguishable (Figures S22–S28). At least at the beginning, the motility kinetics showed quasi-linear behavior for all inhibitors (Figures 3B and S8–S28). The time dependence of the dose–response curves suggested that the results would be most reproducibly assessed after 2 h (Figures 3C,D). At least at low serum concentrations (0–2.5% FBS; Figures S7 and S29−38), single-cell mobility experiments confirmed that the observed area changes originate from motility and not from cell growth. For instance, the movement of single MDA-MB-231 cells on collagen I decelerated from 1.00 ± 0.05 μm min^–1^ (Figure 3E) to 0.40 ± 0.01 μm min^–1^ with 3 μM inhibitor **9** (Figure 3F). This decrease matched the formal wound healing times to close a 1 mm scratch (17 h against 40 h). Because it was important to avoid misinterpretation of cell death as motility inhibition,^56^ CAXs and controls **1**–**12** were reconfirmed^45,51−53^ as nontoxic under the experimental conditions (Figure S3).

Cell migration inhibition by **1**–**16** was assessed for the three cell lines on all three surfaces (Figures S8–S28 and Tables S1–S8). Heatmaps comparing values of IC_50_ or MIC (calculated as IC_15_; see eq S2) were constructed (Figures 3G and S40). The difference between IC_50_ and MIC tracked to the switching half-window C_R_,^57,58^ a metric that can report on cooperativity (large C_R_ indicates negative cooperativity), or on whether several active sites^58^ are involved in an exchange cascade (Figure S41). Fractional heatmaps (Figure 3H,I) and two-component correlations (Figure 3J) were extracted to test for patterns (e.g., inhibitors with similar targets).

Most candidates inhibited the migration of all tested cells on most surfaces, even in the presence of serum (Figures 3G and S8–S37). The selectivity patterns varied between CAXs (heatmap rows), cell types, and surfaces (heatmap columns) (Figure 3G–I). Most CAXs were much more active than the known Ellman reagent **1**.^4^ The inhibition enhancement (IE = IC_50_(**1**)/IC_50_(**test**)) reached ≥70 for chlorodithiabismepane **9**(53) halting the aggressive MDA-MB-231 cells on fibronectin or 32 for the bioinspired epidithiodiketopiperazine (ETP)^51,55^**3** inhibiting MCF-7 cells on the same surface. The typically most efficient CAXs for TMU, such as **9**, **10**, and **3**, gave inhibition that could be up to 10 times more potent than established non-covalent integrin inhibitors such as cyclic RGD **13**,^6,59,60^ α_5_β_1_-selective anti-SARS-CoV-2 **14**,^2,61,62^ and protein disulfide isomerase (PDI) inhibitors **15** and **16** (Figure 3G,H).^63−65^

Fractional heatmaps weighted against ETP **3** highlighted, for instance, its selectivity for MCF-7 cells on all surfaces and the high activity of pnictogen- and tetrel-centered CAXs **8**–**12** against the aggressive MDA-MB-231 cells (Figure 3H). For every cell type on every surface, at least one CAX outperformed the benchmark covalent PDI inhibitor **15**, and the nature of those CAXs varied (exception: HK on F; Figure 3I). Focused two-component correlations are an alternative tool to recognize selectivity, as exemplified here with MDA on F against MCF-7 on V (Figure 3J).

The appearance of unique patterns with distinct hotspots supported that CAX inhibitors operate selectively, beyond global reactivity, with specific mechanisms and therefore have a certain drug discovery potential. The most antimigratory CAXs such as **3**, **9**, and **10** contain highly reactive, fast-exchanging chalcogen-, pnictogen-, and tetrel-centered relays. Those CAXs that are typically excellent for TMU but underperformed in the antimigratory assay were those that (i) produce slow-exchanging, “sticky” dynamic covalent networks that excel for cytosolic delivery, like the bioinspired benzopolysulfane (BPS)^54^**4**, (ii) exchange only in aprotic hydrophobic environments like cyclic thiosulfonate (CTO)^44^**6**, or (iii) may prefer to exchange with other protein partners, such as asparagusic acid (AspA) **2** with the transferrin receptor.^43^ These trends were consistent with the hypothesis that the inhibition of cell motility by the most efficient CAXs operates with fast exchange cascades along thiol or disulfide arrays on cell surfaces.

Good inhibitors of cell motility were overall good inhibitors of thiol-mediated uptake (Figure 4A,B). Comparison with literature IC_50_ for the inhibition of TMU of **17** (ETP **3** with an attached fluorophore)^55^ into HK cells with the motility of MCF-7 and MDA-MB-231 cells on fibronectin gave distinct patterns with weakly linear correlations (dashed lines) that included either all or only chalcogen-centered CAXs. However, these and other low-confidence patterns are not further discussed here, to avoid overinterpretations.

**Figure 4:**
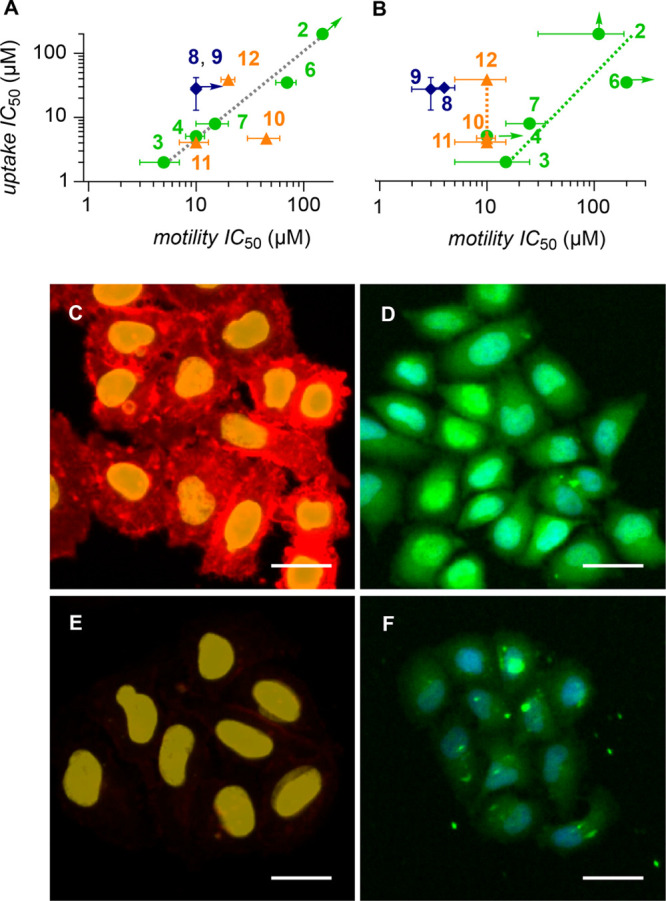
(A) IC_50_ of **2**–**12** for MCF-7 cell motility on fibronectin compared to their IC_50_ for TMU of **17** into HK cells. (B) Similar IC_50_ comparison for MDA-MB-231 cell motility on fibronectin (symbols as in Figure 3J). Uptake data from refs (44), (45), (51)−^53^. (C–F) Fluorescence microscopy images of (C, D) wild-type and (E, F) INT β_1_ siRNA knockdown HK cells incubated with (C, E) immunofluorescence integrin probe (red) and (D, F) **17** (green) (yellow, blue: Hoechst 33342, nuclei; scale bars, 30 μm).

To close the functional feedback loop, β_1_ integrins were knocked down in HK cells, as confirmed by immunofluorescence quantification (Figure 4C vs E). Cellular uptake of fluorescent ETP **17** into the cytosol and mostly the nucleus,^55^ determined under routine conditions,^55^ decreased significantly in the absence of β_1_ integrins (Figure 4D,F). As far as we know, this difference provides the first direct experimental support that integrins act as dynamic covalent exchange partners in thiol-mediated uptake. The complementary motility inhibition could not be measured because the motility of knockdown cells was as poor as expected, reappearing only when integrins were starting to be re-expressed (not shown).

In summary, we report that a focused collection of CAXs inhibits the motility of various cells on various surfaces. Their antimigratory activities exceed that of Ellman’s reagent by far and correlates globally with their abilities to penetrate cells and deliver substrates into the cytosol. Knockdown experiments support the conclusion that the same proteins are involved in TMU as in the antimigratory effect.

This conclusion is important. Despite the rich collection of candidates,^21^ only one TMU partner had previously been robustly identified, i.e., the transferrin receptor, without being general (for AspA,^43^ not ETP^55^). Now we find that the integrin superfamily is the first general exchange partner that has experimental support to participate in TMU. These results thus (a) introduce dynamic covalent cascade exchange chemistry to the control of cell motility, (b) expand the CAX drug discovery space from antiviral toward antithrombotic and antitumor potential, (c) confirm integrins as exchange partners in the dynamic TMU networks that deliver matter into cells, from drugs to pathogens, and thus (d) inspire new design strategies (e.g., multivalent CAXs^30^ to benefit from integrin clustering^66^). These lessons are likely to enable significant and varied future advances.

## Methods

### Automated Cell Motility Inhibition Assay

On a 96-well Black ibiTreat sterile microplate coated with collagen I, fibronectin, or vitronectin (see the Supporting Information), HeLa Kyoto, MCF-7 (6 × 10^4^ cells/well), or MDA-MB-231 cells (9 × 10^4^ cells/well) were seeded in DMEM + 10% FBS and kept overnight at 37 °C under 5% CO_2_ atmosphere. The cell monolayers were scraped with a homemade device (Figure S1) and washed twice with PBS. Then the medium was changed to DMEM (with FBS 0–7.5%), and the inhibitor candidates were added with an electronic multichannel pipet. With an automated confocal microscope, transmitted light (TL) images were recorded at the center of the wells, and time series of 14–26 h were recorded. The onset of toxicity at high inhibitor concentrations was identified by dead rounded-up cells that detached and accumulated in the center of the scratch (Figure S3A), and measurements were limited to concentrations below this threshold (Figure S3B).

Automated analysis processed the original time-lapse TL images (Figure S4A) to generate a relevant mask of the cell layer. The first set of masks determined the cell edge (Figure S4C). This was done by top-hat modification of the TL image that highlights the cell boundaries (Figure S4B). To the segmented image, a size filter was applied to exclude any object below 50 μm^2^ (Figure S4D). Following a similar procedure on a pixel-intensity-inverted image (Figure S5A,B), the cell body was segmented (Figure S5C). Finally, the two cell masks, that is, the cell edge (Figure S4D) and cell body (Figure S5C), were combined to create the cell layer (Figure S6A). This cell layer was then slightly grown to give a homogeneous layer (Figure S6B). Since the growth led to the appearance of some unwanted background objects, a size filter was added to remove all objects below 30 000 μm^2^ to give the final cell layer (Figure S6C). To determine the scratch area, the cell layer (Figure S6C) was subtracted from the whole image mask (including all pixels of the image, Figure S6D) to give the desired area (Figure S6E).

In the final image (Figure S6F), the area of the scratch (*A*) was deduced from the blue area, the cell layer was labeled in yellow, and the space between cells not caused by the scratch (interstitial space) was labeled in cyan. The motility *m* was calculated by subtracting the area of the blue layer at a specific time (*A*_*t*_) from the area at *t* = 0 (*A*_0_), i.e., *m* = *A*_0_ – *A*_*t*_, and normalized against *m*_0_ under the same conditions without inhibitor. Duplicates were performed for each condition and averaged. The relative motility *m*/*m*_0_ was plotted as a function of the inhibitor concentration and fitted to the Hill equation to retrieve IC_15_ (MIC), IC_50_, and *n*.

## Supporting Information Available

Materials and methods; inhibitor synthesis; cell culture; coating procedures; expanded cell motility imaging procedure; expanded AHCHT motility analysis; dependence on inhibitors, concentration, time, cells, surfaces, and serum; pattern generation; single-cell motility inhibition procedure; ITGB1 knockdown; cellular uptake inhibition procedure (PDF)
Video of AHCHT motility assay with inhibitor **9** (MPG)
Video of AHCHT motility assay without inhibitor (MPG)

## Supplementary Material

au3c00113_si_001.pdf

au3c00113_si_002.mpg

au3c00113_si_003.mpg
