# Supplementary material for: Inhibition of Cell Motility by Cell-Penetrating Dynamic Covalent Cascade Exchangers: Integrins Participate in Thiol-Mediated Uptake
Source: JACS Au. 2023 Apr 12;3(4):1010–6. doi: 10.1021/jacsau.3c00113 (PMC10131202; doi:10.1021/jacsau.3c00113)
Supplement: Supplementary file 1 — au3c00113_si_001.pdf [file au3c00113_si_001.pdf]

Supporting Information

Inhibition of Cell Motility by Cell-Penetrating Dynamic Covalent Cascade Exchangers: Integrins Participate in Thiol-Mediated Uptake

Filipe Coelho,[†,‡] Saidbakhrom Saidjalolov,[†,‡] Dimitri Moreau,[‡] Oliver Thorn-Seshold,[§] and Stefan Matile*,[†,‡]

[†]Department of Organic Chemistry, University of Geneva, CH-1211 Geneva, Switzerland

[‡]Department of Biochemistry, University of Geneva, CH-1211 Geneva, Switzerland

[§]Department of Pharmacy, Ludwig-Maximilians University of Munich, 81377 Munich, Germany

*E-mail: stefan.matile@unige.ch

Table of Contents

1.	Materials and Methods		S4
2.	Synthesis		S5
	2.1.	Synthesis of Inhibitors	S5
	2.2.	Synthesis of Transporter	S6
3.	Cell Culture		S6
4.	Automated High-Content High-Throughput (AHCHT) Imaging Assay		S6
	4.1.	Coating Procedure	S6
	4.2.	General Procedure for AHCHT Motility Inhibitor Screening	S7
	4.3.	Data Analysis for AHCHT Imaging of Cell Motility	S8
	4.4.	Dependence of Cell Motility on Serum Concentration	S13
5.	MDA-MB-231 Cell Motility		S13
	5.1.	Collagen	S14
	5.2.	Fibronectin	S19
	5.3.	Vitronectin	S22
6.	MCF-7 Cell Motility		S23
	6.1.	Collagen	S24
	6.2.	Fibronectin	S29
	6.3.	Vitronectin	S32
7.	HeLa Kyoto Cell Motility		S35
	7.1.	Collagen	S36
	7.2.	Fibronectin	S41
	7.3.	Vitronectin	S44
8.	Inhibition of Cell Motility in the Presence of Serum		S47
	8.1.	MDA-MB-231 Cells	S48

8.2. MCF-7 Cells S52

8.3. HeLa Kyoto Cells S56

8.4. Heatmap S60

9. Single Cell Motility S61

9.1. General Procedure for Single Cell Motility Inhibition S61

9.2. Data Analysis for Single Cell Motility Inhibition S61

10. Knockdown of ITGB1 S62

10.1. General Procedure for the Knockdown of ITGB1 in HK Cells S62

10.2. General Procedure for the Uptake in Knocked-down HK Cells S62

10.3. General Procedure for Integrin Knockdown Quantification by Immunofluorescence S62

11. Pattern Generation S63

12. Supporting References S66

1. Materials and Methods

As mentioned in ref. S1, phosphate buffered saline (PBS, pH = 7.4), DMEM (GlutaMAX, 4.5 g/L D-glucose, with phenol red) medium, Penicillin- Streptomycin, RNAiMAX, Fetal Bovine Serum, TrypLE Express Enzyme and V96-MicroWell plate were obtained from Thermo Fisher Scientific. μ-Plate 96-Well was obtained from Ibidi. Hoechst 33342 (10 mg/mL solution in water) was obtained from Invitrogen by Thermo Fisher Scientific. Collagen I was obtained from Med. Cruz. Vitronectin and allicin were obtained from MedChem Express. Cyclic-RGD, DTNB, Fibronectin, ATN-161, 16F16 and PACMA-31 were obtained from Sigma-Aldrich. The siRNA against INT B1 was obtained from siPOOL (NCBI Gene ID 3688). The lipofectamine RNAiMAX was obtained from Invitrogen (13778-030). The reduced serum medium Opti-MEM was obtained from Gibco. The anti-integrin β1 antibody was obtained from abcam (mouse monoclonal, ab24693). The secondary antibody was obtained from Jackson Immuno LTD. (715-605-150). Imaging was performed using an IXM-C automated microscope from imageXpress equipped with a Lumencor Aura III with 5 independent selectable solid-state light sources, bandpass filters and 5 objectives (4x to 60x). Cells were scraped using 200 μL sterile tips with filter from Starlab held by hand or using a house made scratching tool (Figure S1). Washing steps were performed using a plate washer Biotek EL406®.

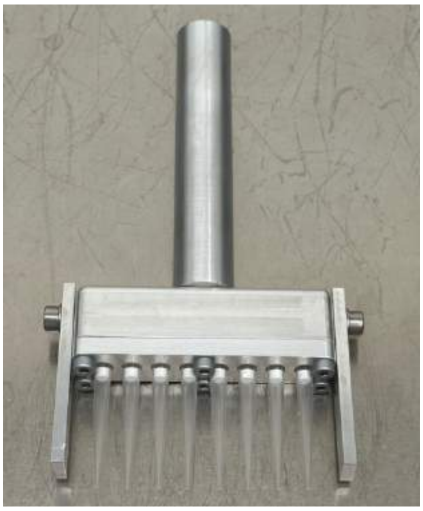

Figure S1. Tool used to scratch μ-Plate 96-Well.

Abbreviations. AspA: Asparagusic acid; AHCHT: Automated high-content high-throughput; BPS: Benzopolysulfane; C: Collagen I; CAX: Covalent exchangers; CTO: Cyclic thiosulfonate; DMEM: Dulbecco's Modified Eagle Medium; DTNB: 5,5'-Dithiobis-(2- nitrobenzoic acid); ETP: Epidithiodiketopiperazine; F: Fibronectin; FBS: Fetal bovine serum; HK: HeLa Kyoto; MAC: Michael acceptor; MIC: Minimum inhibitory concentration; PBS: Phosphate-buffered saline; PDI: Protein disulfide isomerase; rt: Room temperature; TMU: Thiol-mediated uptake; V: Vitronectin.

2. Synthesis

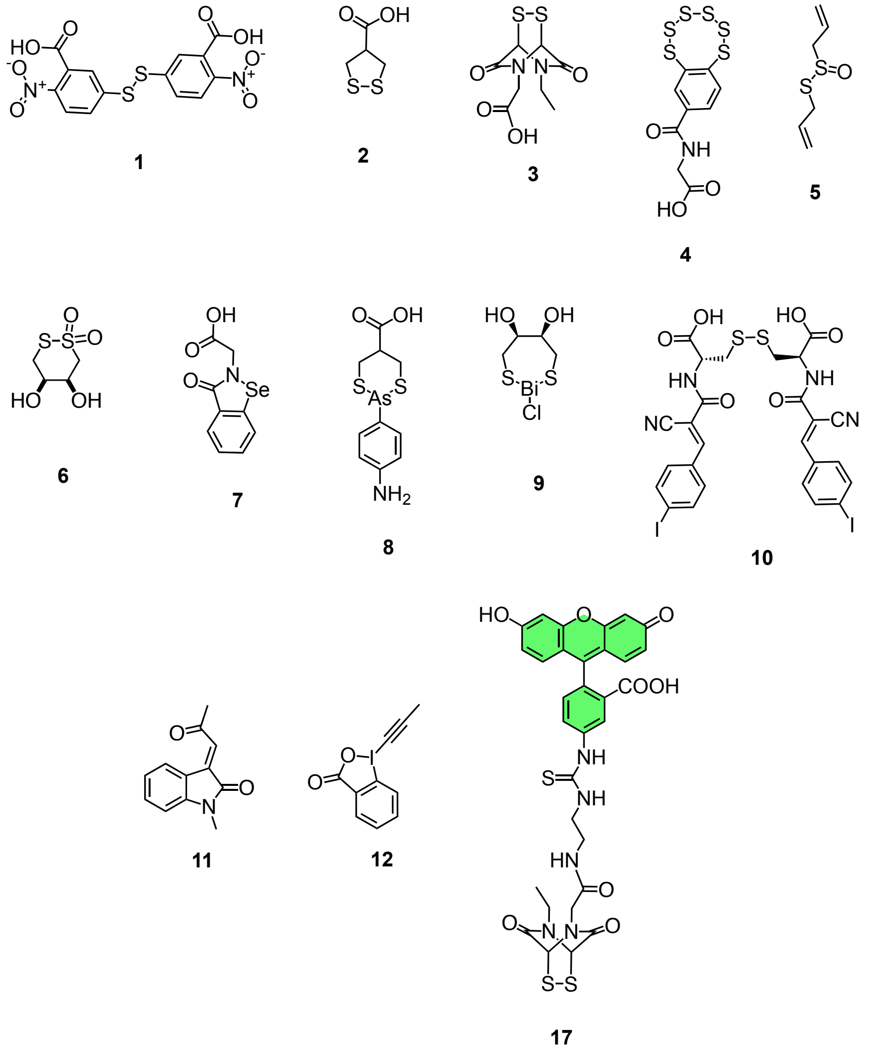

Figure S2. Structures of inhibitors and a reporter.

2.1. Synthesis of Inhibitors

Compounds 2, **3**, **4**, and **6** were synthesized according to procedures described in ref. S2.

Compound 7 was synthesized according to procedures described in ref. S3.

Compounds 8 and **9** were synthesized according to procedures described in ref. S4.

Compound 10 was synthesized according to procedures described in ref. S1.

Compound 11 was synthesized according to procedures described in ref. S5.

Compound 12 was synthesized according to procedures described in ref. S6.

2.2. Synthesis of Transporter

Compounds 17 was synthesized according to procedures described in ref. S7.

3. Cell Culture

Human cervical cancer-derived HeLa Kyoto, human breast cancer-derived MCF-7 and MDA-MB-231 cells were cultured in DMEM (GlutaMAX, 4.5 g/L D-glucose, with phenol red) medium containing 10% fetal bovine serum (FBS) and 1% Penicillin/Streptomycin (PS). The cells were grown at 37 °C under 5% CO_2 on a 75 cm^3 tissue culture flask (TPD Corporation). Cells were detached by treatment with 3 mL of TrypLE Express at 37 °C for 5 min, followed by the addition of 10 mL of DMEM (GlutaMAX, 4.5 g/L D-glucose, with phenol red) medium at 37 °C. The cells were spun down at 1000 g for 5 min, resuspended in DMEM (GlutaMAX, 4.5 g/L D-glucose, with phenol red) medium, and plated according to the concentration needed.

4. Automated High-Content High-Throughput (AHCHT) Imaging Assay

4.1. Coating Procedure

Collagen I. Coating protocol was provided by ibidi. Briefly, a solution of collagen I (50 μg/mL in ddH_2O) was pipetted into a μ-Plate 96-well Black ibiTreat sterile (200 μL/well) and left to incubate at room temperature for 1 h. Finally, the solution was aspirated, and the cells were immediately seeded.

Fibronectin. Coating protocol was obtained from ibidi. Briefly, a solution of fibronectin (10 μg/mL in PBS) was pipetted to a μ-Plate 96-well Black ibiTreat sterile (100 μL/well) and left to

incubate at room temperature for 1 h. Finally, the solution was aspirated, and the cells were immediately seeded.

Vitronectin. Coating protocol was obtained from Sigma-Aldrich. Briefly, a solution of vitronectin (3 µg/mL in PBS) was pipetted to a µ-Plate 96-well Black ibiTreat sterile (100 µL/well) and left to incubate for 1 h at 37 °C under 5% CO_2 atmosphere. Then, the plate was kept in a fridge (4 °C) overnight. The next morning, the solution was aspirated, and the cells were immediately seeded.

4.2. General Procedure for AHCHT Motility Inhibitor Screening

HeLa Kyoto and MCF-7 cells were seeded at 6 $\times 10^4$ cells/well and MDA-MB-231 cells were seeded at 9 $\times 10^4$ cells/well in DMEM + 10% FBS on coated µ-Plate 96-well Black ibiTreat sterile and kept at 37 °C under 5% CO_2 atmosphere overnight. Next day, each well was scratched horizontally with a 200 µL micropipette tip leaving a scratch of around 1 mm. The cells were washed with PBS (1 × 2 mL/well) and the medium was exchanged to DMEM (with FBS 0-7.5%, 4 x 150 µL), using a plate washer while keeping a final volume of 135 µL/well. The washing protocol was run twice for each plate. The serial dilutions of the inhibitors were prepared in FluoroBrite DMEM in a 96-well V-bottom plate. The inhibitor solutions from the V-bottom plate were added to the cells (15 µL/well) using an electronic multichannel pipette to reach a final volume of 150 µL/well, and the plate was imaged using an automated confocal microscope. For each experiment, a transmitted light (TL) image at the center of the well was acquired at 4× (3.5 × 3.5 mm). A time series of 14-26 h was acquired in duplicates. Onset of toxicity at high inhibitor concentrations was visually identifiable since dead round up cells detach and accumulate in the center of the scratch (Figure S3A), while in healthy conditions a homogenous cell layer with very few rounded cells (dividing mostly) can be observed (B). Wells containing generalized detachment of cells were not taken into consideration for the motility inhibition assay.

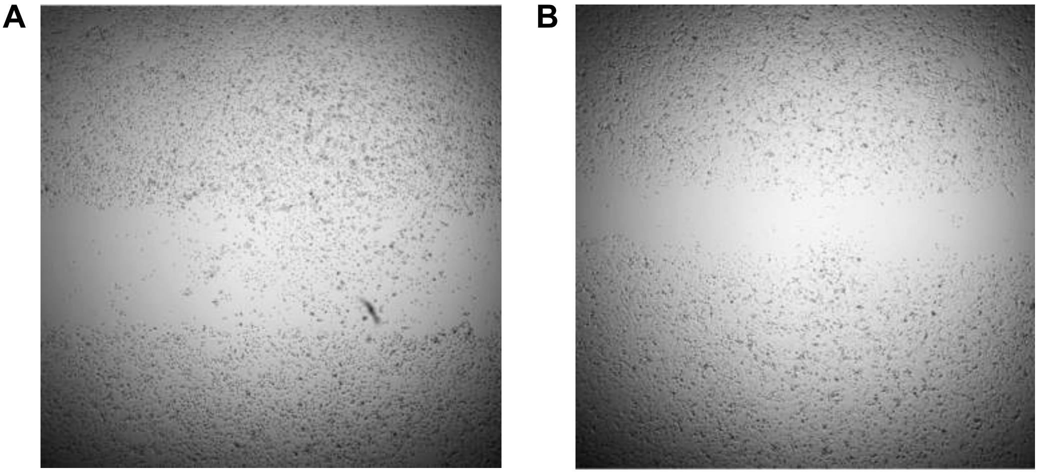

Figure S3. Evidence of toxicity in MDA-MB-231 cells caused by high concentration of inhibitor **12** (30 μM) (A). Healthy MDA-MD-231 cells (B).

4.3. Data Analysis for AHCHT Imaging of Cell Motility

The microscope acquires time lapse TL image per well over a period of time (Figure S4A). Each image was then run through a series image processing and modification to generate relevant mask of the cell layer. The first set of masks determined the cell edge (Figure S4C). This was done by top hat modification of the TL image that highlights the cell boundaries (Figure S4B). A size filter was then applied to the segmented image to exclude any object below 50 μm^2 to remove unwanted debris (Figure S4D).

Following a similar procedure on a pixel intensity inverted image (Figures S5A and S5B) the cell body was segmented (Figure S5C).

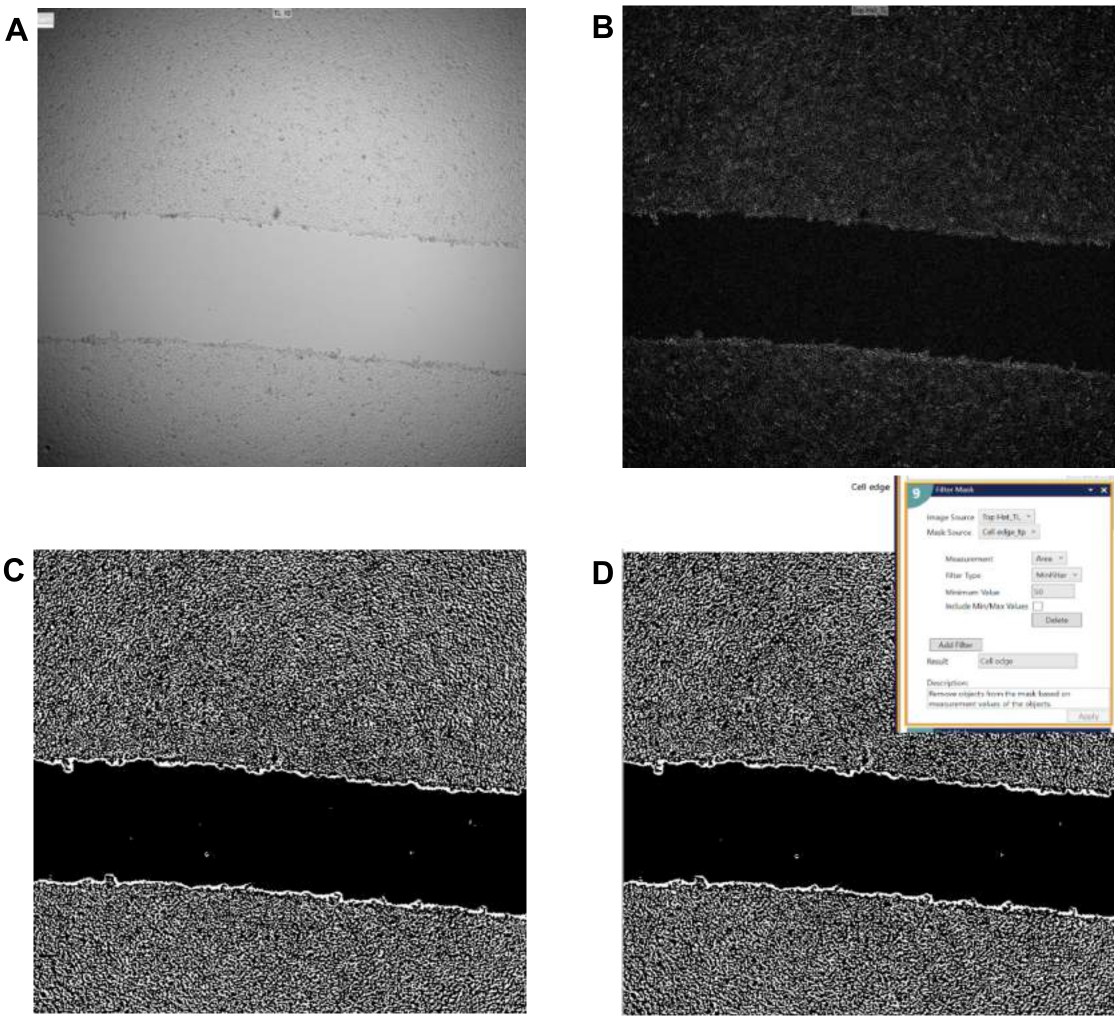

Figure S4. TL (A) and top hat (B) images. Cell boundaries could be clearly observed in white on the inverted image. Segmentation of cell edge (C) and filtering of objects by size (excluding < 50 μm^2, D).

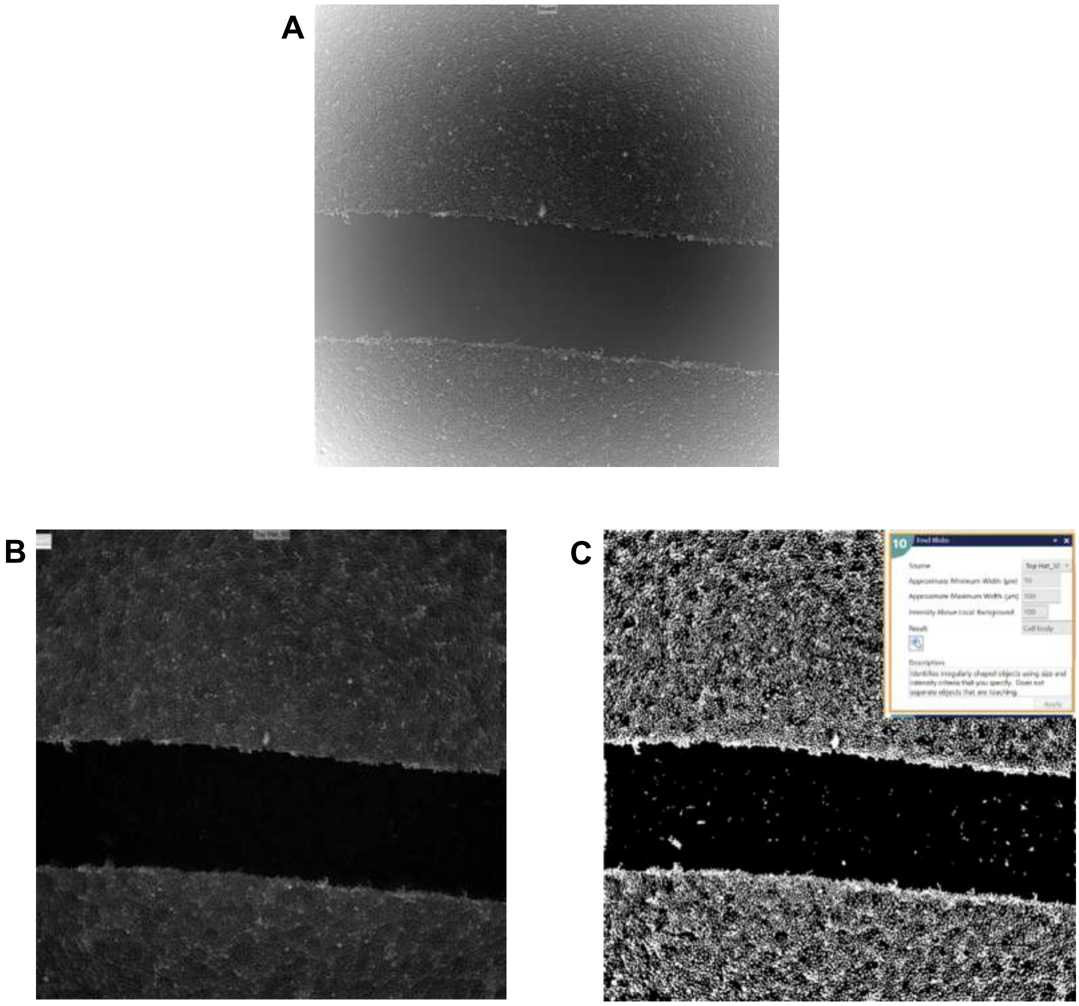

Figure S5. Inverted TL image (A), top hat mask applied to highlight the cell body (B) and creation of cell body objects by segmentation (C).

Finally, both cell masks – cell edge (Figure S4D) and cell body (Figure S5C) – were combined to create *cell layer* (Figure S6A). The cell layer was then slightly grown to give a homogenous layer (Figure S6B). Since the growth led to the appearance of some unwanted background objects, a size filter was used to remove all objects below 30 000 μm^2 to give the final cell layer (Figure S6C).

To determine the scratch area, the cell layer (Figure S6C) was subtracted to the whole image mask (including all pixels of the image, Figure S6D) to give the desired area (Figure S6E). On the final image (Figure S6F), the area of the scratch (A) was deduced from the blue area, the cell layer was labeled in yellow and the space between cells not caused by the scratch (interstitial space) was labeled in cyan. The motility (m) was calculated by subtracting the area at $t = 0$ (A_0) to the area of the blue layer at a specific time (A_t) using Equation (S1), and then normalized against m obtained under the conditions without inhibitor (m_0). Duplicates were performed for each condition and averaged.

$$m = A_0 - A_\mathrm{t} \tag{S1}$$

The resulting relative motility m/m_0 was plotted as a function of the inhibitor concentration and fitted with Equation (S2) to retrieve the half maximal inhibitory concentration (IC_{50}) and the Hill coefficient (n). MIC values were estimated from the curve fit as the concentration at which 15% of inhibition was obtained.

$$m/m_0 = 1 \, / \, (1 + (\mathrm{IC}_{50} \, / \, c_{inhibitor})^{-n}) \tag{S2}$$

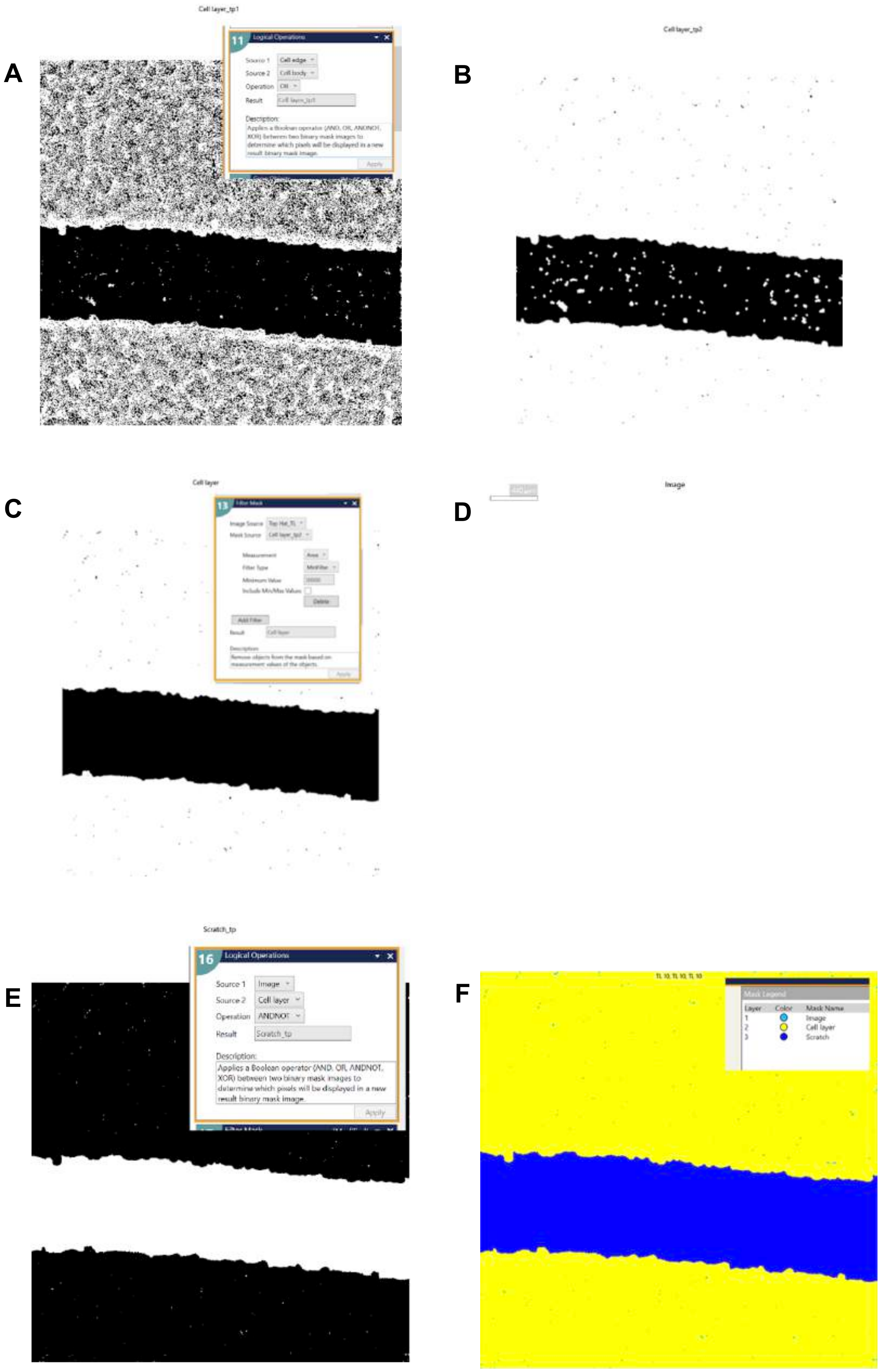
A
Cell layer_tp1
Logical Operations
Source 1
Cell edge
Source 2
Cell body
Operation
OR
Result
Description:
B
Cell layer_tp2
C
Cell layer
Filter Mask
D
Image
E
Scratch_tp
16
Logical Operations
Source 1
Image
Source 2
Cell layer
Operation
ANDNOT
Result
Scratch_tp
Description:
Applies a Boolean operator (AND, OR, ANDNOT, XOR) between two binary mask images to determine which pixels will be displayed in a new result binary mask image.
Apply
F
Mask Legend
Layer
Color
Mask Name
1
Image
2
Cell layer
3
Scratch

Figure S6. Cell layer image obtained from combining cell edge and body (A), growth of objects to create homogeneous layer (B), elimination of unwanted objected created by growth (C), white image with same number of pixels as the original image (D), scratch obtained from subtraction of cell layer and the blank image (E), final image containing the quantification of the different elements, cell layer (yellow), scratch (blue) and interstitial space (cyan, F).

4.4. Dependence of Cell Motility on Serum Concentration

The presence of serum in culture media favors fast cell division, which in this case, will push cells into the scratch faster than in medium deprived of serum. This effect can produce a false sense of motility. With this in mind, inhibition measurements were set at 0% serum. Measuments in collagen were also conducted in 2.5% serum as a control.

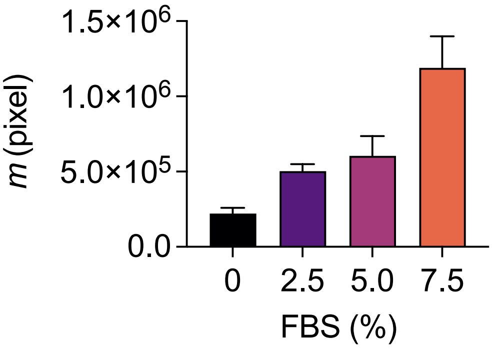

Figure S7. Dependence of migration on percentage of fetal bovine serum (FBS) evaluated in HK cells on collagen following the protocol described in 4.2. The motility was followed for 16 h.

5. MDA-MB-231 Cell Motility

The motility inhibition was measured according to the procedure described in 4.2. and analyzed as described in 4.3. The masks obtained for each coating are shown in Figures S8 (C), S11 (F) and S13 (V). Application of Equation (S1) afforded the motility, which was plotted at a fixed concentration over time as shown in Figures S9 (C), S12 (F) and S14 (V). The relative motility (m/m_0) at different concentrations was calculated to plot dose-response curves (Figure S10). The fit of these curves to Equation (S2) afforded parameters such as the concentration needed to reach 50% inhibition

(IC_{50}), the concentration needed to reach 15% inhibition (MIC) and the Hill coefficient (*n*) for inhibition of cellular motility (Tables S1 (C) and S2 (F)).

5.1. Collagen

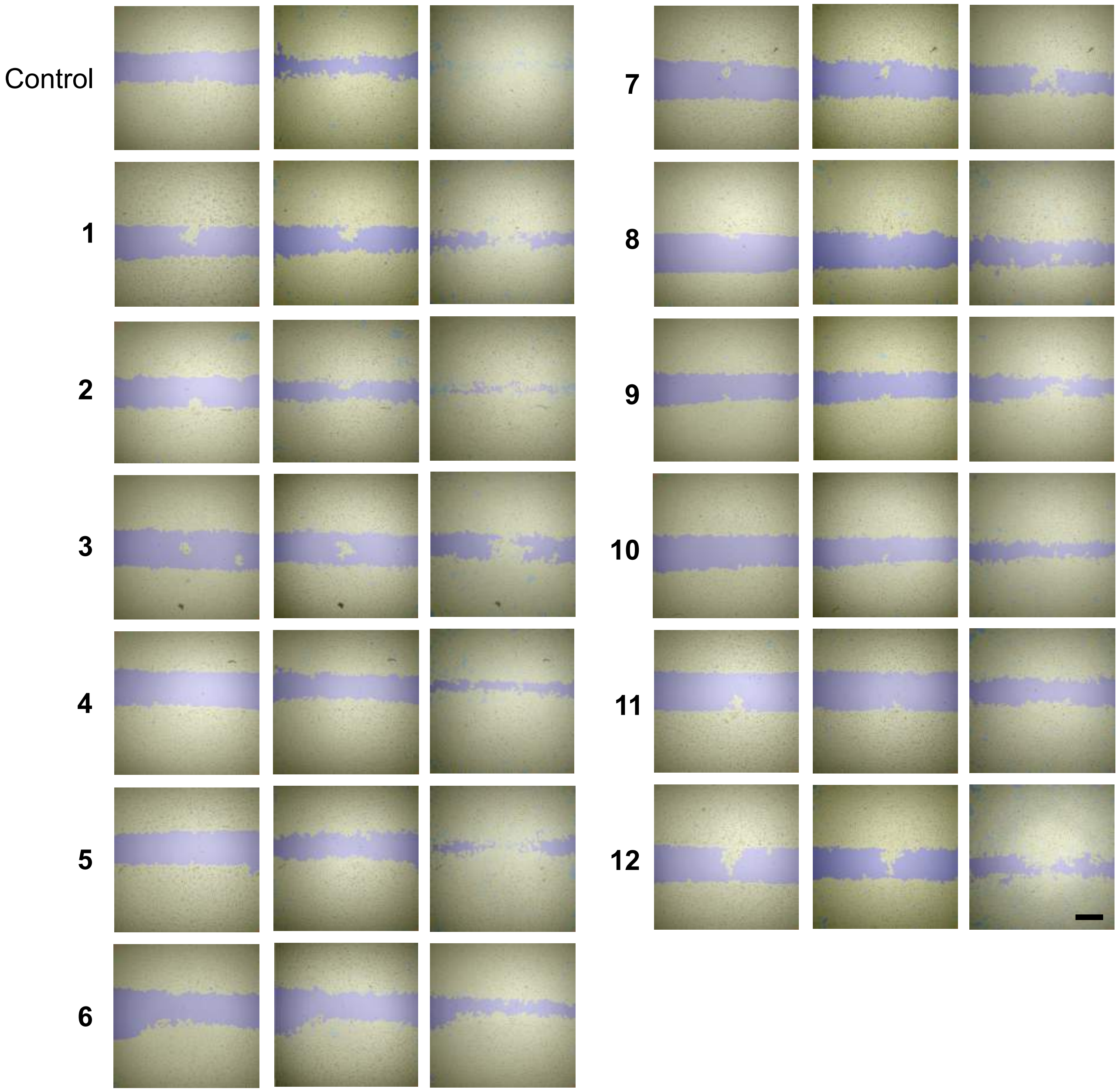

Figure S8. AHCHT images with overlayed masks of MDA-MB-231 cells on collagen taken at 1 h (left), 8 h (middle) and 20 h (right) in the presence of **1** (200 µM), **2** (150 µM), **3** (30 µM), **4** (17 µM), **5** (50 µM), **6** (200 µM), **7** (50 µM), **8** (3 µM), **9** (3 µM), **10** (50 µM), **11** (20 µM) and **12** (5 µM). Scale bar: 1 mm.

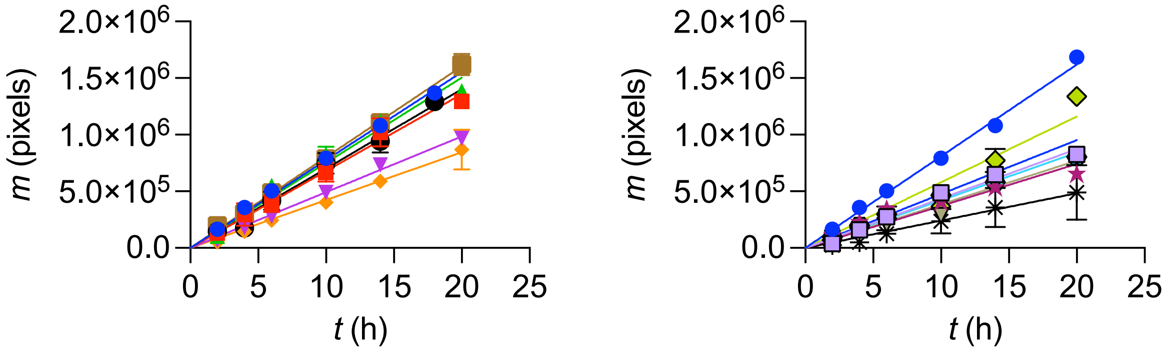

Figure S9. Motility as a function of time for MDA-MB-231 cells on collagen I. (A) Control (dark blue circles), **1** (red squares, 200 µM), **2** (green triangles, 150 µM), **3** (purple inverted triangles, 30 µM), **4** (orange diamonds, 17 µM), **5** (black circles, 50 µM), **6** (brown squares, 200 µM). (B) Control (dark blue, circles), **7** (purple squares, 50 µM), **8** (bordeaux stars, 3 µM), **9** (olive inverted triangles, 3 µM), **10** (lime diamonds, 50 µM), **11** (black asterisks, 20 µM) and **12** (light blue hexagons, 5 µM).

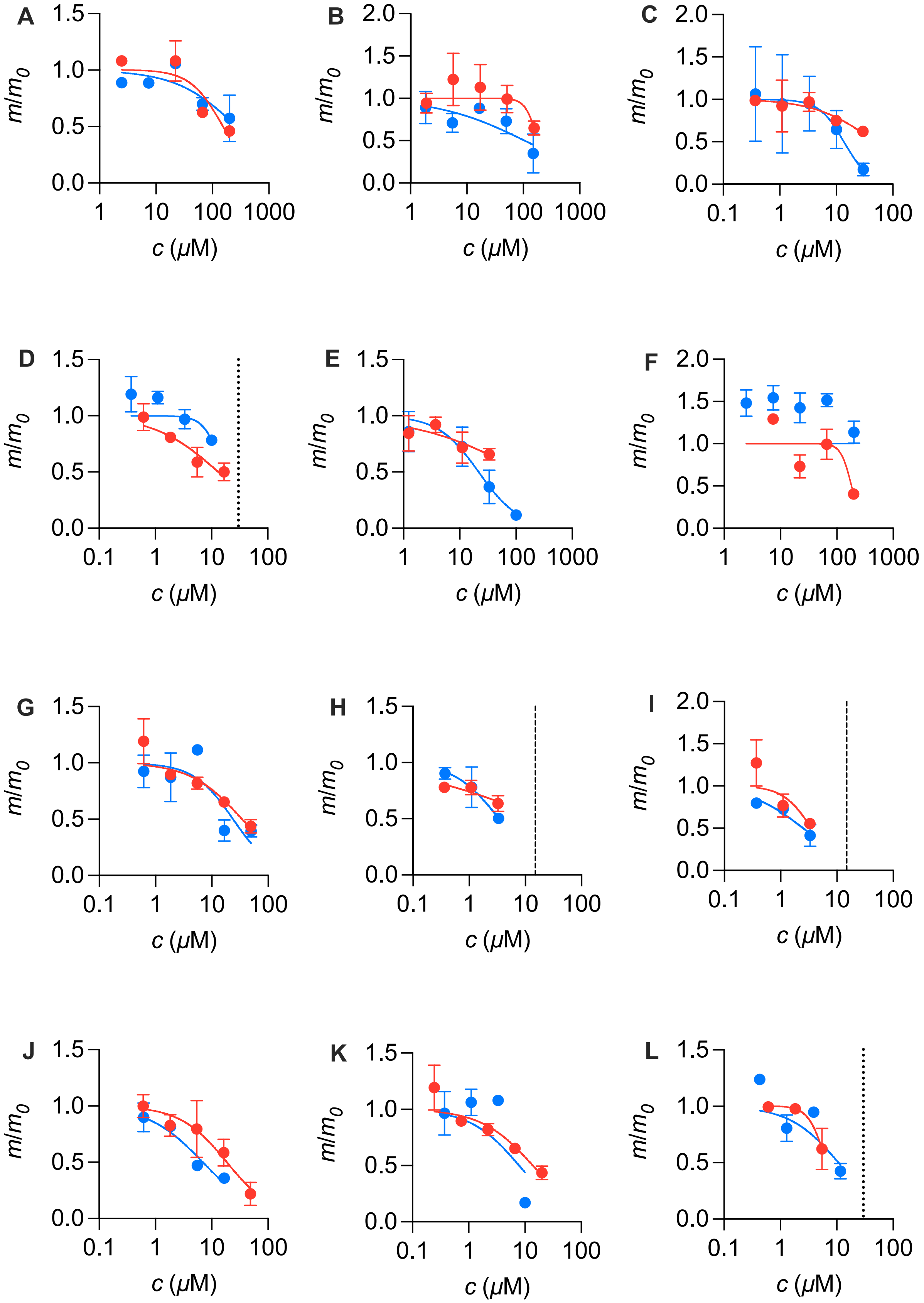
A
B
C
D
E
F
G
H
I
J
K
L
m/m0
c (μM)

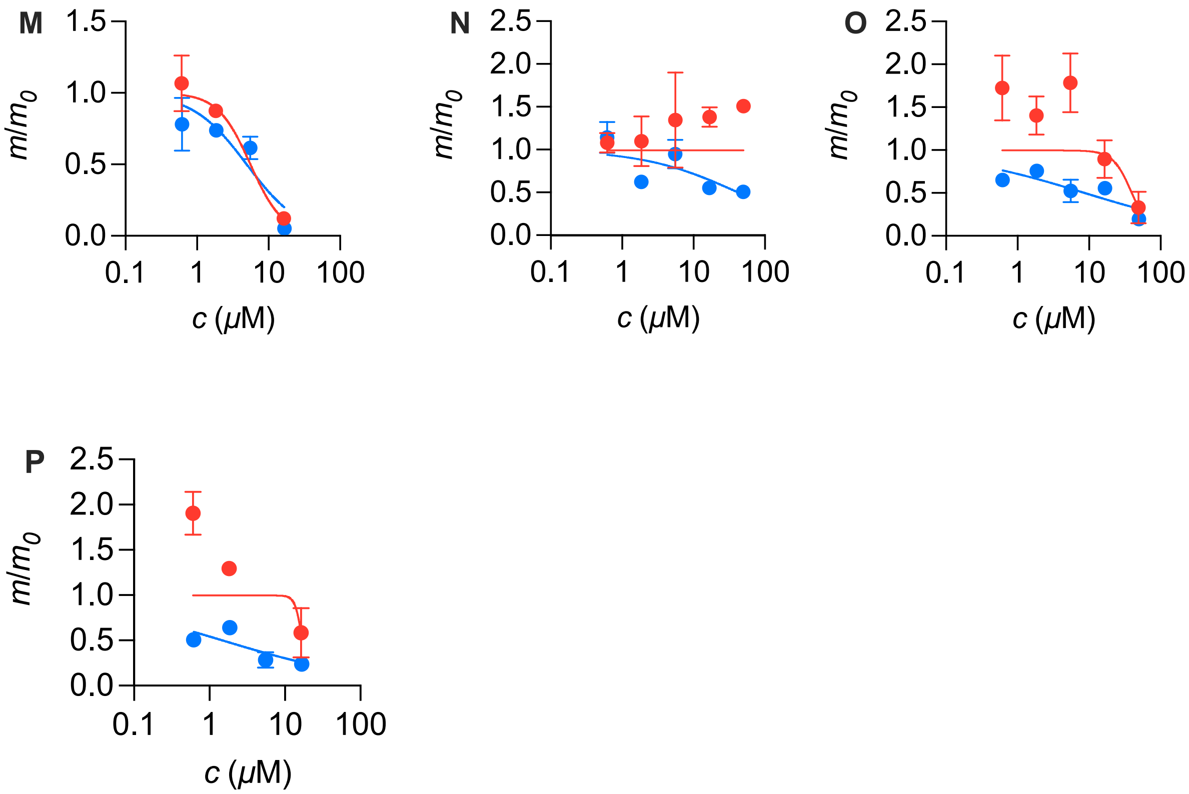

Figure S10. AHCHT data showing relative motility m/m_0 ± SEM of MDA-MB-231 on collagen I (red) and fibronectin (blue) coated plates after 2 h of incubation as a function of the concentration of A) **1**, B) **2**, C) **3**, D) **4**, E) **5**, F) **6**, G) **7**, H) **8**, I) **9**, J) **10**, K) **11**, L) **12**, M) **13**, N) **14**, O) **15** and P) **16**. Dashed (c = 15 µM) and dotted lines (c = 30 µM) represent the concentration at which the onset of toxicity was observed.

Table S1. Inhibition of MDA-MB-231 cell motility on collagen I.[a]

Entry	I[b]	MIC (µM)[c]	IC_{50} (µM)[d]	*n*[e]
1	**1**	50	150 ± 70	2 ± 1
2	**2**	110	>150	-
3	**3**	5	30 ± 15	0.8 ± 0.6
4	**4**	1	15 ± 5	0.8 ± 0.3
5	**5**	3	>50	-
6	**6**	130	>200	-
7	**7**	10	35 ± 15	0.9 ± 0.4
8	**8**	<0.4	>3	-
9	**9**	2	4 ± 2	1.3 ± 0.7
10	**10**	5	20 ± 7	1.0 ± 0.4
11	**11**	2	15 ± 6	1.0 ± 0.4
12	**12**	5	10 ± 3	3 ± 1
13	**13**	2	5 ± 2	2 ± 1
14	**14**	-	-	-
15	**15**	20	40 ± 25	3 ± 1
16	**16**	15	20 ±7	9 ± 4

[a]Data obtained after 2 h of incubation. [b]Inhibitors. [c]Concentration needed to reach 15% inhibition. [d]Concentration needed to reach 50% inhibition (best fit ± SEM). [e]Hill coefficient for inhibition of cellular motility (best fit ± SEM). Data corresponding to the dose response curves presented in Figure S10.

5.2. Fibronectin

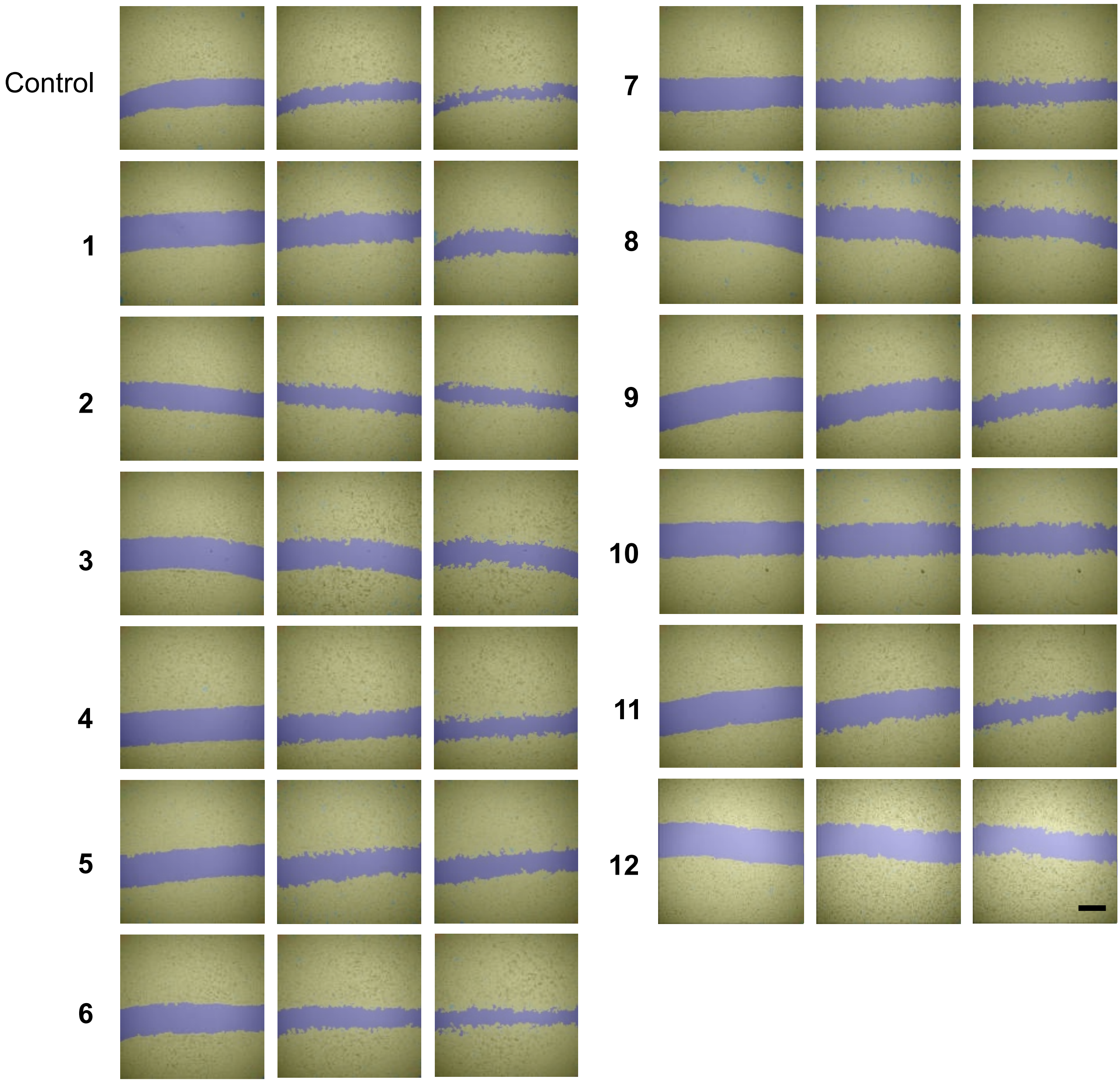

Figure S11. AHCHT images with overlayed masks of MDA-MB-231 cells on fibronectin taken at 1 h (left), 8 h (middle) and 14 h (right) in the presence of **1** (200 µM), **2** (150 µM), **3** (30 µM), **4** (10 µM), **5** (100 µM), **6** (200 µM), **7** (50 µM), **8** (3 µM), **9** (3 µM), **10** (17 µM), **11** (10 µM) and **12** (10 µM). Scale bar: 1 mm.

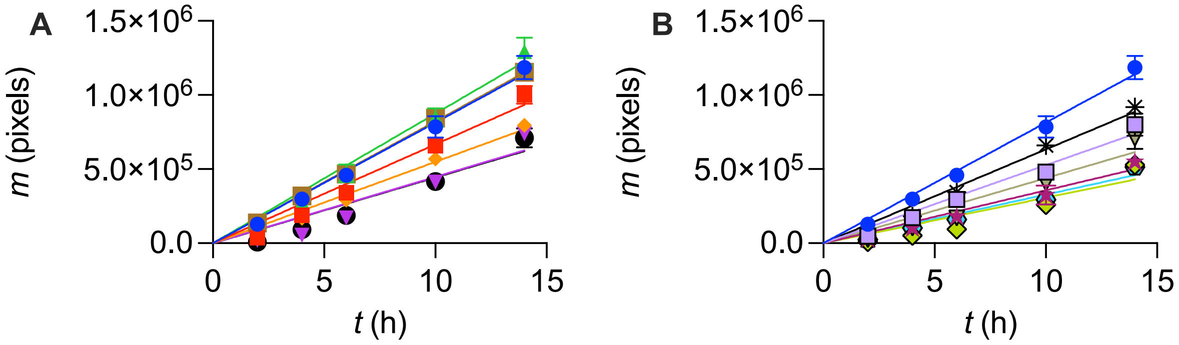

Figure S12. Motility as a function of time for MDA-MB-231 cells on fibronectin coated surface. (A) Control (dark blue circles), **1** (red squares, 200 μM), **2** (green triangles, 150 μM), **3** (purple inverted triangles, 30 μM), **4** (orange diamonds, 10 μM), **5** (black circles, 100 μM), **6** (brown squares, 200 μM). (B) **7** (purple squares, 50 μM), **8** (bordeaux stars, 3 μM), **9** (olive inverted triangles, 3 μM), **10** (lime diamonds, 17 μM), **11** (black asterisks, 10 μM) and **12** (light blue hexagons, 10 μM).

Table S2. Inhibition of MDA-MB-231 cell motility on fibronectin.[a]

Entry	I[b]	MIC (µM)[c]	IC_{50} (µM)[d]	n[e]
1	**1**	30	>200	-
2	**2**	20	110 ± 80	0.6 ± 0.3
3	**3**	6	15 ± 10	2 ± 1
4	**4**	8	>10	-
5	**5**	5	20 ± 7	1.0 ± 0.5
6	**6**	-	-	-
7	**7**	10	25 ± 10	1.2 ± 0.6
8	**8**	1	4 ± 1	0.6 ± 0.4
9	**9**	<0.4	3 ± 1	0.6 ± 0.3
10	**10**	<0.6	10 ± 2	0.9 ± 0.3
11	**11**	1	10 ± 5	1.0 ± 0.7
12	**12**	5	10 ± 5	1.0 ± 0.6
13	**13**	1	5 ± 2	1.1 ± 0.4
14	**14**	5	40 ± 30	0.6 ± 0.3
15	**15**	<0.4	10 ± 5	0.4 ± 0.1
16	**16**	<0.4	2 ± 1	0.4 ± 0.2

[a]Data obtained after 2 h of incubation. [b]Inhibitors. [c]Concentration needed to reach 15% inhibition. [d]Concentration needed to reach 50% inhibition (best fit ± SEM). [e]Hill coefficient for inhibition of cellular motility (best fit ± SEM). Data corresponding to the dose response curves presented in Figure S10.

5.3. Vitronectin

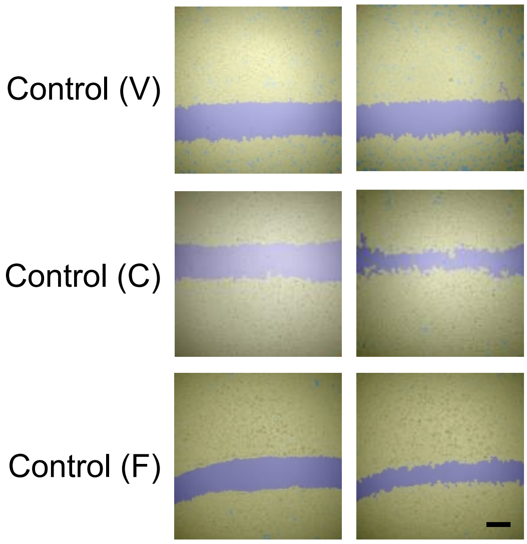

Figure S13. AHCHT images with overlayed masks of MDA-MB-231 cells on vitronectin (V), collagen I (C) and fibronectin (F) taken at 1 h (left) and 8 h (right). Scale bar: 1 mm.

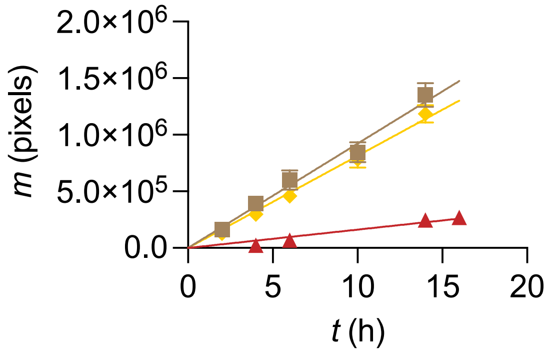

Figure S14. Motility as a function of time for MDA-MB-231 cells on vitronectin (red triangles), collagen I (brown squares) and fibronectin (yellow diamonds). Motility of these cells on vitronectin is very poor due to the lack of $\alpha_v\beta_3$ integrins. Therefore, inhibition experiments were not meaningful.

6. MCF-7 Cell Motility

The motility inhibition was measured according to the procedure described in 4.2. and analyzed as described in 4.3. The masks obtained for each coating are shown in Figures S15 (C), S18 (F) and S20 (V). Application of Equation (S1) afforded the motility, which was plotted at a fixed concentration over time as shown in Figures S16 (C), S19 (F) and S21 (V). The relative motility (m/m_0) at different concentrations was calculated to plot dose-response curves (Figure S17). The fit of these curves to Equation (S2) afforded parameters such as the concentration needed to reach 50% inhibition (IC_{50}), the concentration needed to reach 15% inhibition (MIC) and the Hill coefficient (n) for inhibition of cellular motility (Tables S3 (C), S4 (F) and S5 (V)).

6.1. Collagen

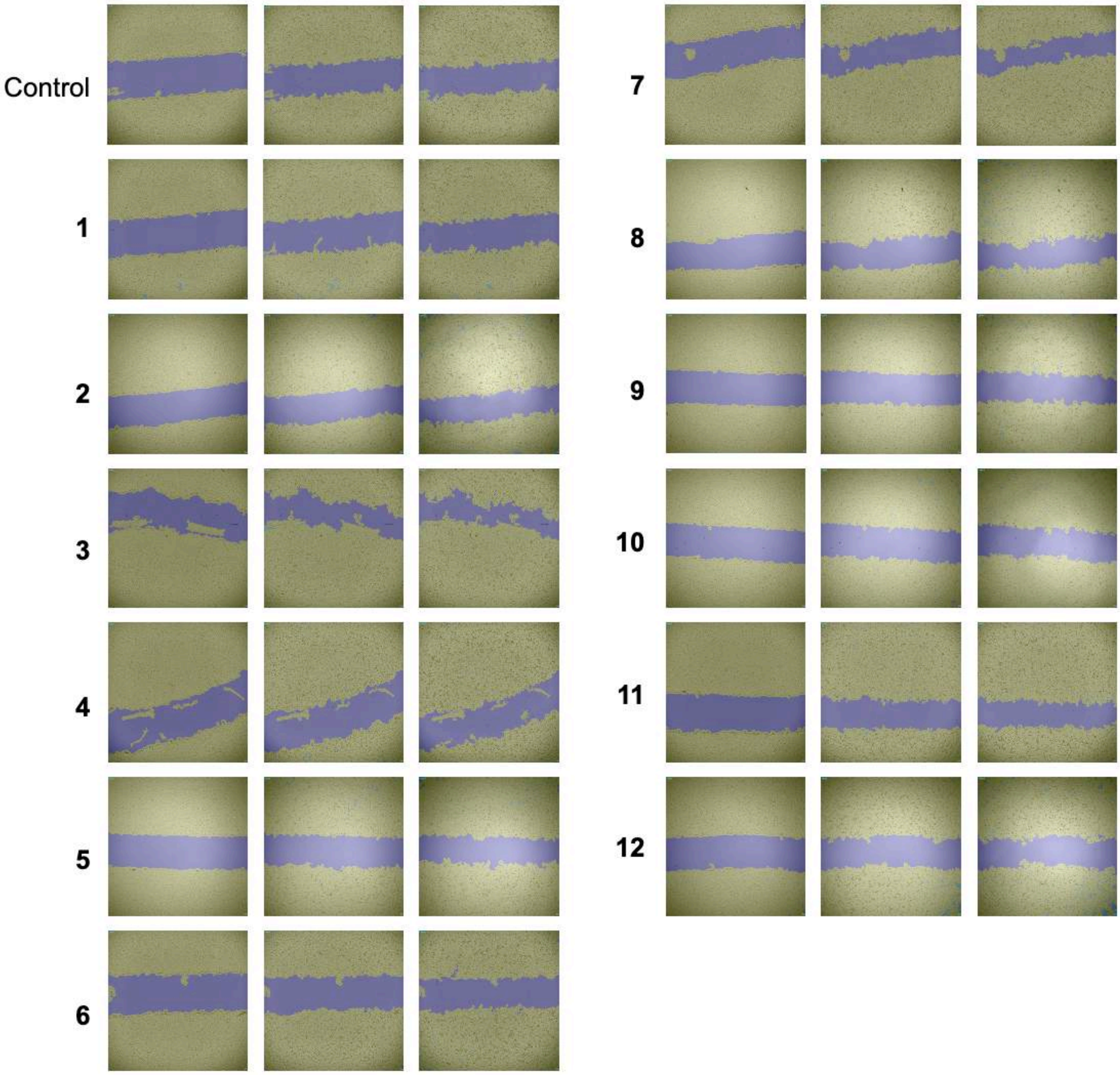

Figure S15. AHCHT images with overlayed masks of MCF-7 cells on collagen taken at 1 h (left), 8 h (middle) and 16 h (right) in the presence of **1** (200 μM), **2** (150 μM), **3** (12 μM), **4** (17 μM), **5** (75 μM), **6** (200 μM), **7** (17 μM), **8** (3 μM), **9** (3 μM), **10** (50 μM), **11** (30 μM) and **12** (10 μM). Scale bar: 1 mm.

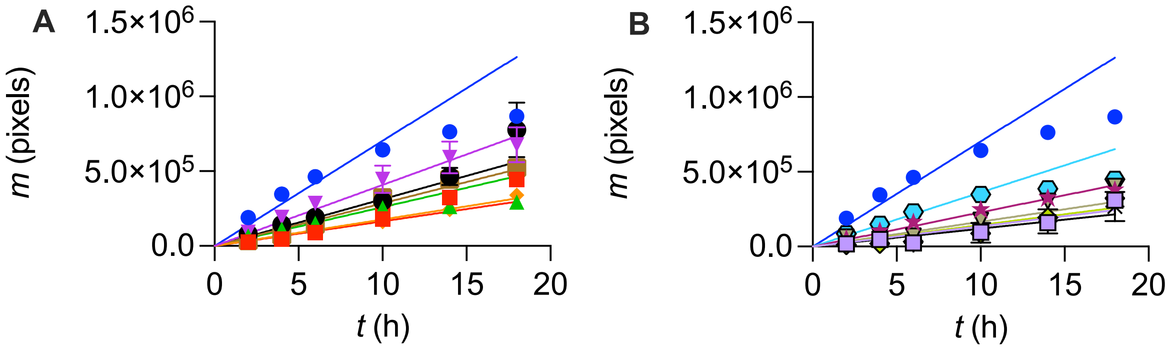

Figure S16. Motility as a function of time for MCF-7 cells on collagen I. (A) Control (dark blue circles), **1** (200 μM, red squares), **2** (150 μM, green triangles), **3** (12 μM, purple inverted triangles), **4** (17 μM, orange diamonds), **5** (75 μM, black circles), **6** (200 μM, brown squares). (B) Control (dark blue circles), **7** (17 μM, purple squares), **8** (3 µM, bordeaux stars), **9** (3 µM, olive inverted triangles), **10** (50 μM, lime diamonds), **11** (30 μM, black asterisks) and **12** (10 μM, light blue hexagons).

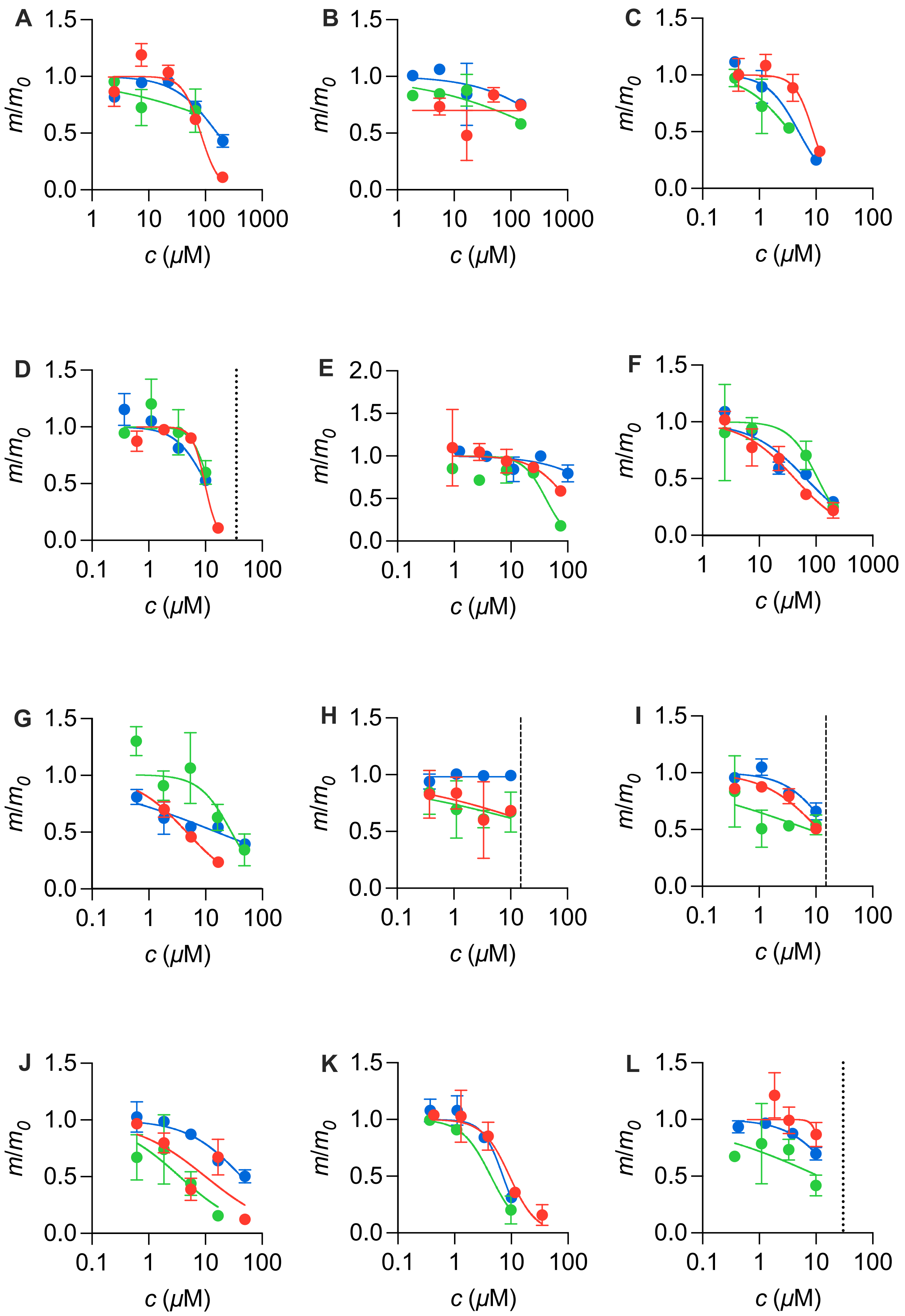
A
B
C
D
E
F
G
H
I
J
K
L
m/m0
c (μM)
0.0
0.5
1.0
1.5
2.0
0.1
1
10
100
1000

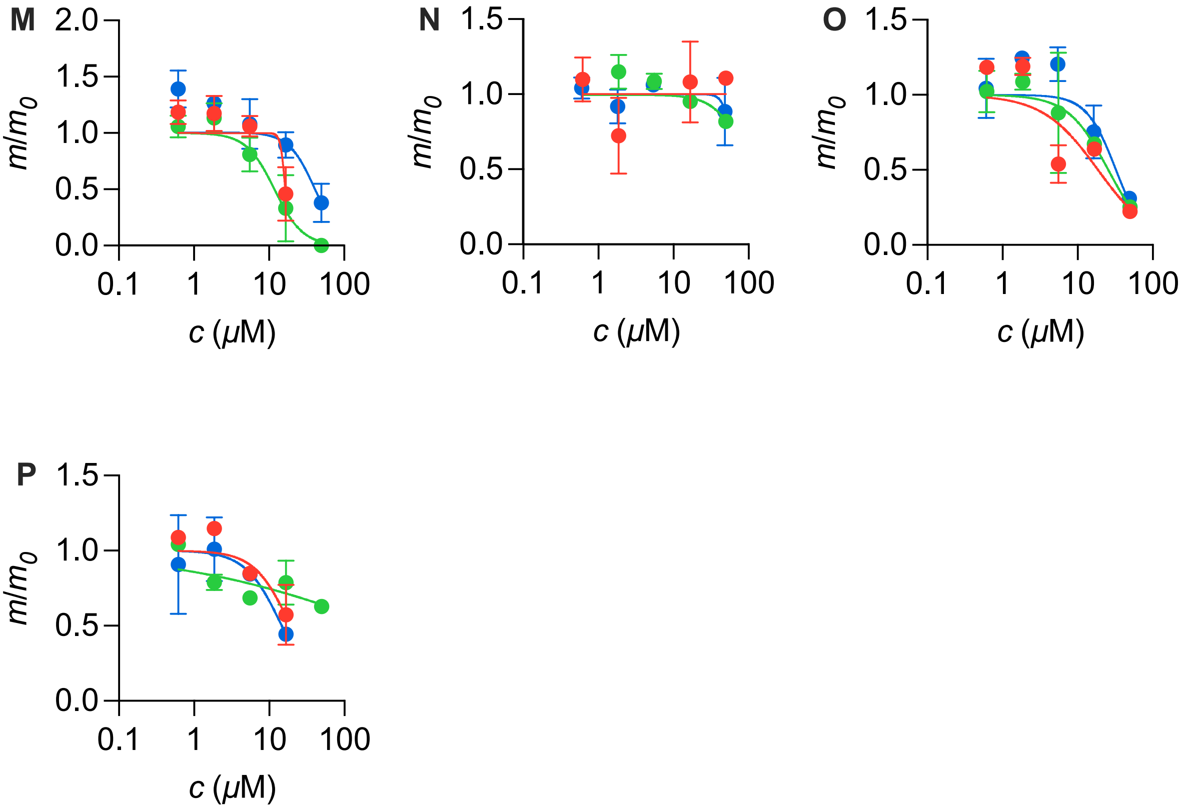

Figure S17. AHCHT data showing relative motility m/m_0 ± SEM of MCF-7 cells on collagen I (red), fibronectin (blue) and vitronectin (green) coated plates after 2 h of incubation as a function of the concentration of A) **1**, B) **2**, C) **3**, D) **4**, E) **5**, F) **6**, G) **7**, H) **8**, I) **9**, J) **10**, K) **11**, L) **12**, M) **13**, N) **14**, O) **15** and P) **16**. Dashed (c = 15 µM) and dotted lines (c = 30 µM) represent the concentration at which the onset of toxicity was observed.

Table S3. Inhibition of MCF-7 cell motility on collagen I. [a]

Entry	I[b]	MIC (μM)[c]	IC_{50} (μM)[d]	n[e]
1	**1**	40	80 ± 10	1.0 ± 0.5
2	**2**	-	-	-
3	**3**	5	10 ± 2	3 ± 1
4	**4**	6	10 ± 6	1.0 ± 0.5
5	**5**	30	>75	-
6	**6**	7	40 ± 10	0.9 ± 0.2
7	**7**	<0.6	4 ± 1	1.0 ± 0.2
8	**8**	<0.4	>10	-
9	**9**	<0.4	>10	
10	**10**	1	10 ± 5	1.0 ± 0.3
11	**11**	3	10 ± 2	1.0 ± 0.3
12	**12**	5	>10	-
13	**13**	14	>17	-
14	**14**	-	-	-
15	**15**	4	20 ± 6	1.2 ± 0.5
16	**16**	7	>17	-

[a]Data obtained after 2 h of incubation. [b]Inhibitors. [c]Concentration needed to reach 15% inhibition. [d]Concentration needed to reach 50% inhibition (best fit ± SEM). [e]Hill coefficient for inhibition of cellular motility (best fit ± SEM). Data corresponding to the dose response curves presented in Figure S17.

6.2. Fibronectin

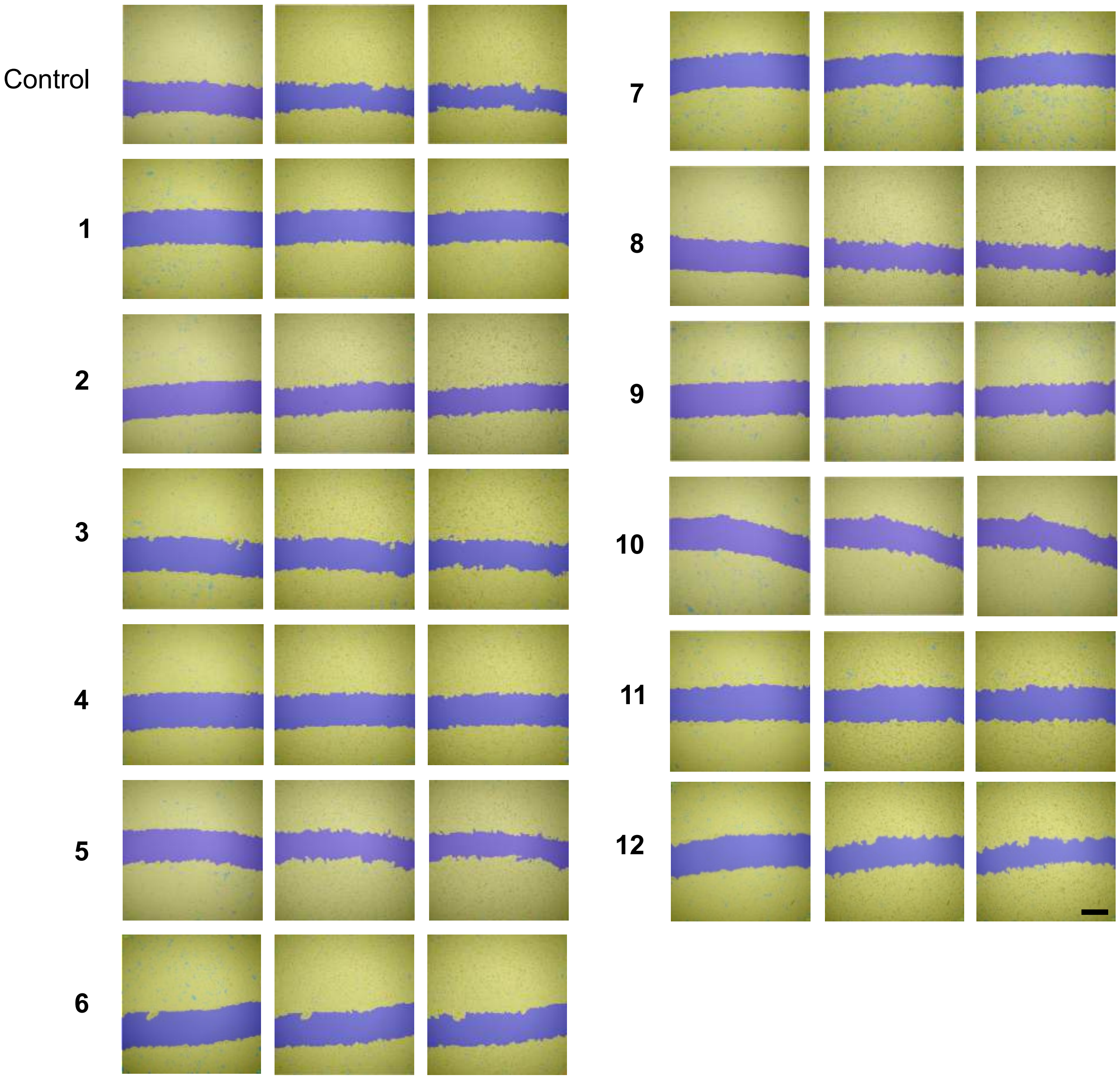

Figure S18. AHCHT images with overlayed masks of MCF-7 cells on fibronectin taken at 1 h (left), 8 h (middle) and 14 h (right) in the presence of **1** (200 µM), **2** (150 µM), **3** (10 µM), **4** (10 µM), **5** (100 µM), **6** (200 µM), **7** (50 µM), **8** (3 µM), **9** (3 µM), **10** (50 µM), **11** (10 µM) and **12** (10 µM). Scale bar: 1 mm.

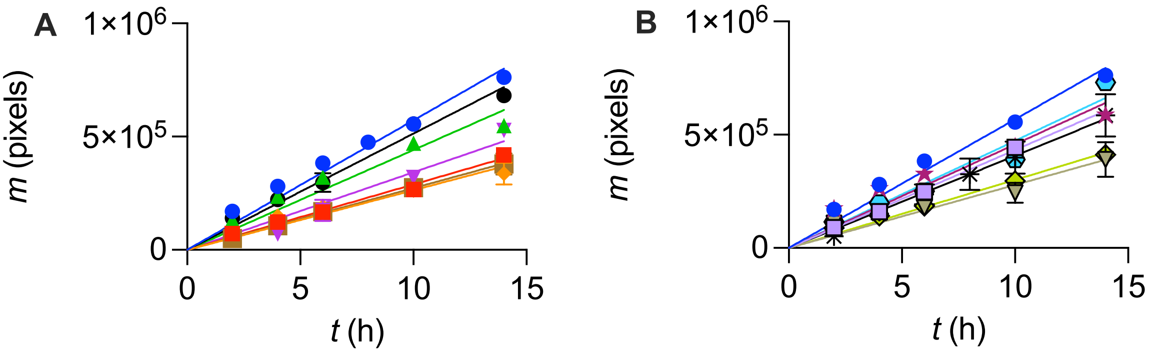

Figure S19. Motility as a function of time for MCF-7 cells on fibronectin. (A) Control (dark blue circles), **1** (200 μM, red squares), **2** (150 μM, green triangles), **3** (10 μM, purple inverted triangles), **4** (10 μM, orange diamonds), **5** (100 μM, black circles), **6** (200 μM, brown squares). (B) Control (dark blue circles), **7** (50 μM, purple squares), **8** (3 µM, bordeaux stars), **9** (3 µM, olive inverted triangles), **10** (50 μM, lime diamonds), **11** (10 μM, black asterisks) and **12** (10 μM, light blue hexagons).

Table S4. Inhibition of MCF-7 cell motility on fibronectin. [a]

Entry	I[b]	MIC (μM)[c]	IC_{50} (μM)[d]	n^e
1	**1**	40	160 ± 40	1.1 ± 0.3
2	**2**	50	>150	-
3	**3**	2	5 ± 2	1.6 ± 0.5
4	**4**	5	10 ± 2	2 ± 1
5	**5**	70	>100	-
6	**6**	5	70 ± 15	0.9 ± 0.2
7	**7**	<0.6	15 ±5	0.3 ± 0.1
8	**8**	-	>10	-
9	**9**	5	>10	
10	**10**	6	45 ± 15	0.9 ± 0.3
11	**11**	5	10 ± 3	2 ± 1
12	**12**	5	20 ± 3	1.4 ± 0.3
13	**13**	20	40 ± 10	3 ± 2
14	**14**	-	>50	-
15	**15**	15	30 ± 20	2 ± 1
16	**16**	5	15 ± 5	2 ± 1

[a]Data obtained after 2 h of incubation. [b]Inhibitors. [c]Concentration needed to reach 15% inhibition. [d]Concentration needed to reach 50% inhibition (best fit ± SEM). [e]Hill coefficient for inhibition of cellular motility (best fit ± SEM). Data corresponding to the dose response curves presented in Figure S17.

6.3. Vitronectin

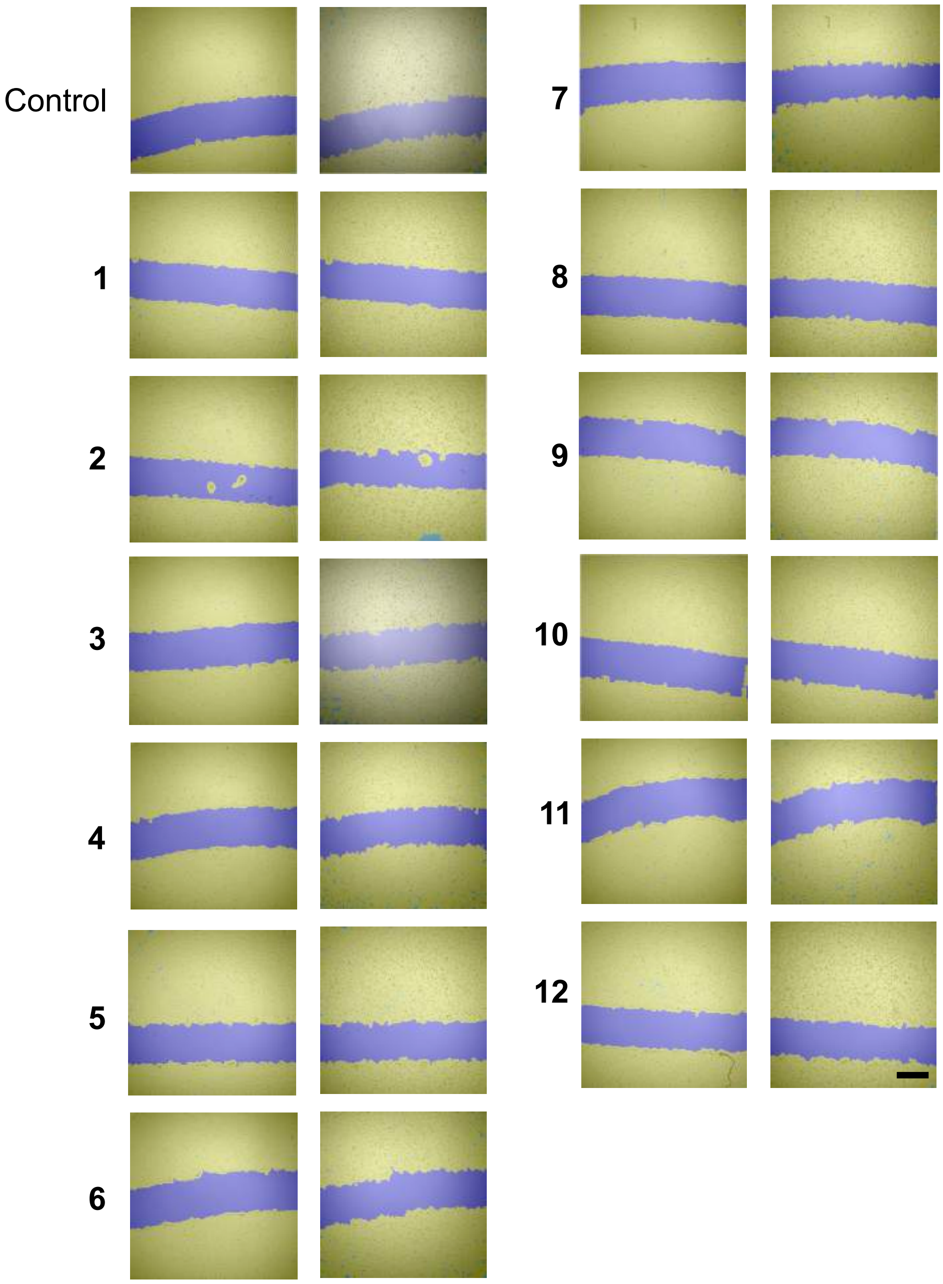

Figure S20. AHCHT images with overlayed masks of MCF cells on vitronectin taken at 1 h (left), 10 h (right) in the presence of **1** (67 µM), **2** (150 µM), **3** (5 µM), **4** (10 µM), **5** (75 µM), **6** (200 µM), **7** (50 µM), **8** (3 µM), **9** (3 µM), **10** (17 µM), **11** (10 µM) and **12** (10 µM). Scale bar: 1 mm.

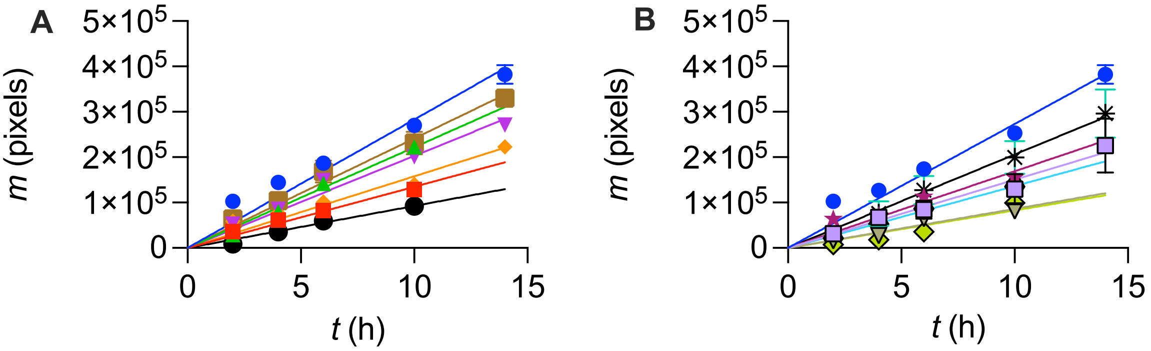

Figure S21. Motility as a function of time for MCF-7 cells on vitronectin. (A) Control (dark blue circles), **1** (200 μM, red squares), **2** (150 μM, green triangles), **3** (5 μM, purple inverted triangles), **4** (10 μM, orange diamonds), **5** (75 μM, black circles), **6** (200 μM, brown squares). (B) Control (dark blue circles), **7** (50 μM, purple squares), **8** (3 µM, bordeaux stars), **9** (3 µM, olive inverted triangles), **10** (17 μM, lime diamonds), **11** (10 μM, black asterisks) and **12** (10 μM, light blue hexagons).

Table S5. Inhibition of MCF-7 cell motility on vitronectin. [a]

Entry	I[b]	MIC (µM)[c]	IC_{50} (µM)[d]	n[e]
1	**1**	4	>67	-
2	**2**	10	>150	-
3	**3**	1	4 ± 2	1.1 ± 0.6
4	**4**	6	10 ± 3	3 ± 1
5	**5**	20	40 ± 25	2 ± 2
6	**6**	40	110 ± 80	2 ± 2
7	**7**	10	30 ± 10	2 ± 1
8	**8**	<0.4	>10	-
9	**9**	<0.4	8 ± 2	0.4 ± 0.2
10	**10**	1	4 ± 1	1 ± 1
11	**11**	2	4 ± 2	2 ± 0.6
12	**12**	<0.4	10 ± 4	0.4 ± 0.1
13	**13**	5	10 ± 3	2 ± 1
14	**14**	-	-	-
15	**15**	10	30 ± 10	2 ± 1
16	**16**	1	>50	-

[a]Data obtained after 2 h of incubation. [b]Inhibitors. [c]Concentration needed to reach 15% inhibition. [d]Concentration needed to reach 50% inhibition (best fit ± SEM). [e]Hill coefficient for inhibition of cellular motility (best fit ± SEM). Data corresponding to the dose response curves presented in Figure S17.

7. HeLa Kyoto Cell Motility

The motility inhibition was measured according to the procedure described in 4.2. and analyzed as described in 4.3. The masks obtained for each coating are shown in Figures S22 (C), S25 (F) and S27 (V). Application of Equation (S1) afforded the motility, which was plotted at a fixed concentration overtime as shown in Figures S23 (C), S26 (F) and S28 (V). The relative motility (m/m_0) at different concentrations was calculated to plot dose-response curves (Figure S24). The fit of these curves to Equation (S2) afforded parameters such as the concentration needed to reach 50% inhibition (IC_{50}), the concentration needed to reach 15% inhibition (MIC) and the Hill coefficient (n) for inhibition of cellular motility (Tables S6 (C), S7 (F) and S8 (V)).

7.1. Collagen

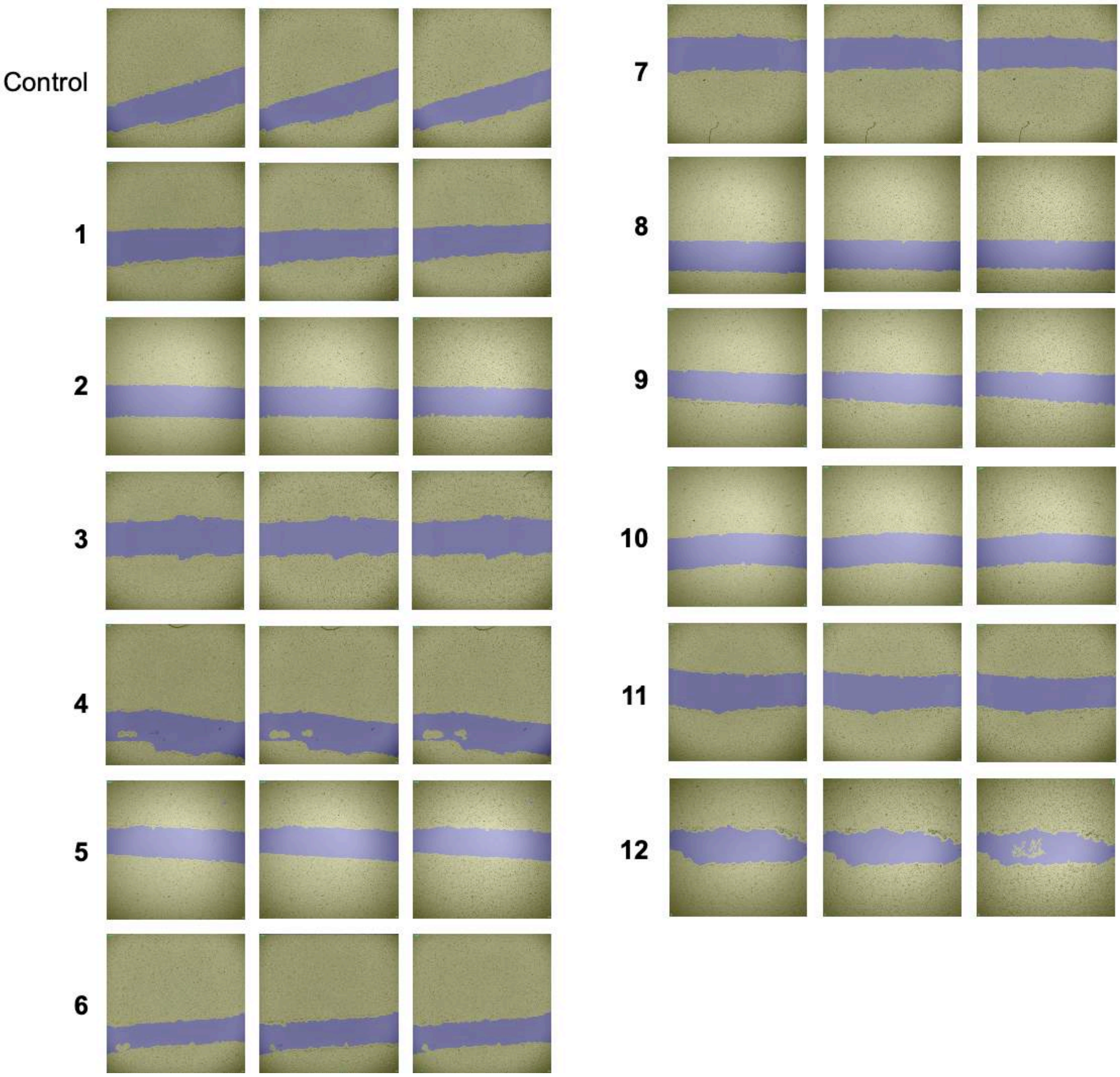

Figure S22. AHCHT images with overlayed masks of HK cells on collagen I taken at 1 h (left), 8 h (middle) and 14 h (right) in the presence of **1** (200 µM), **2** (150 µM), **3** (35 µM), **4** (30 µM), **5** (100 µM), **6** (200 µM), **7** (50 µM), **8** (3 µM), **9** (3 µM), **10** (17 µM), **11** (30 µM) and **12** (35 µM). Scale bar: 1 mm.

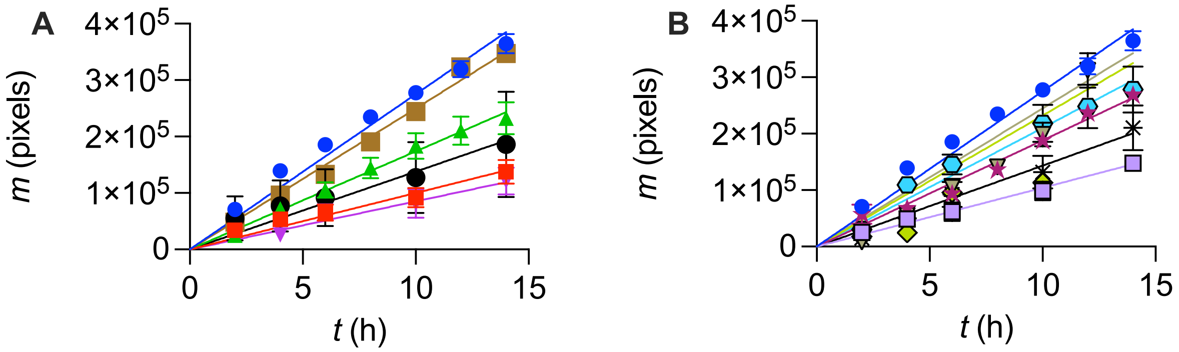

Figure S23. Motility as a function of time for HK cells on collagen I. (A) Control (dark blue circles), **1** (red squares, 200 µM), **2** (green triangles, 150 µM), **3** (purple inverted triangles, 35 µM), **4** (orange diamonds, 30 µM), **5** (black circles, 100 µM), **6** (brown squares, 200 µM). (B) Control (dark blue circles), **7** (purple squares, 10 µM), **8** (bordeaux stars, 3 µM), **9** (olive inverted triangles, 3 µM), **10** (lime diamonds, 17 µM), **11** (black asterisks, 30 µM) and **12** (light blue hexagons, 35 µM).

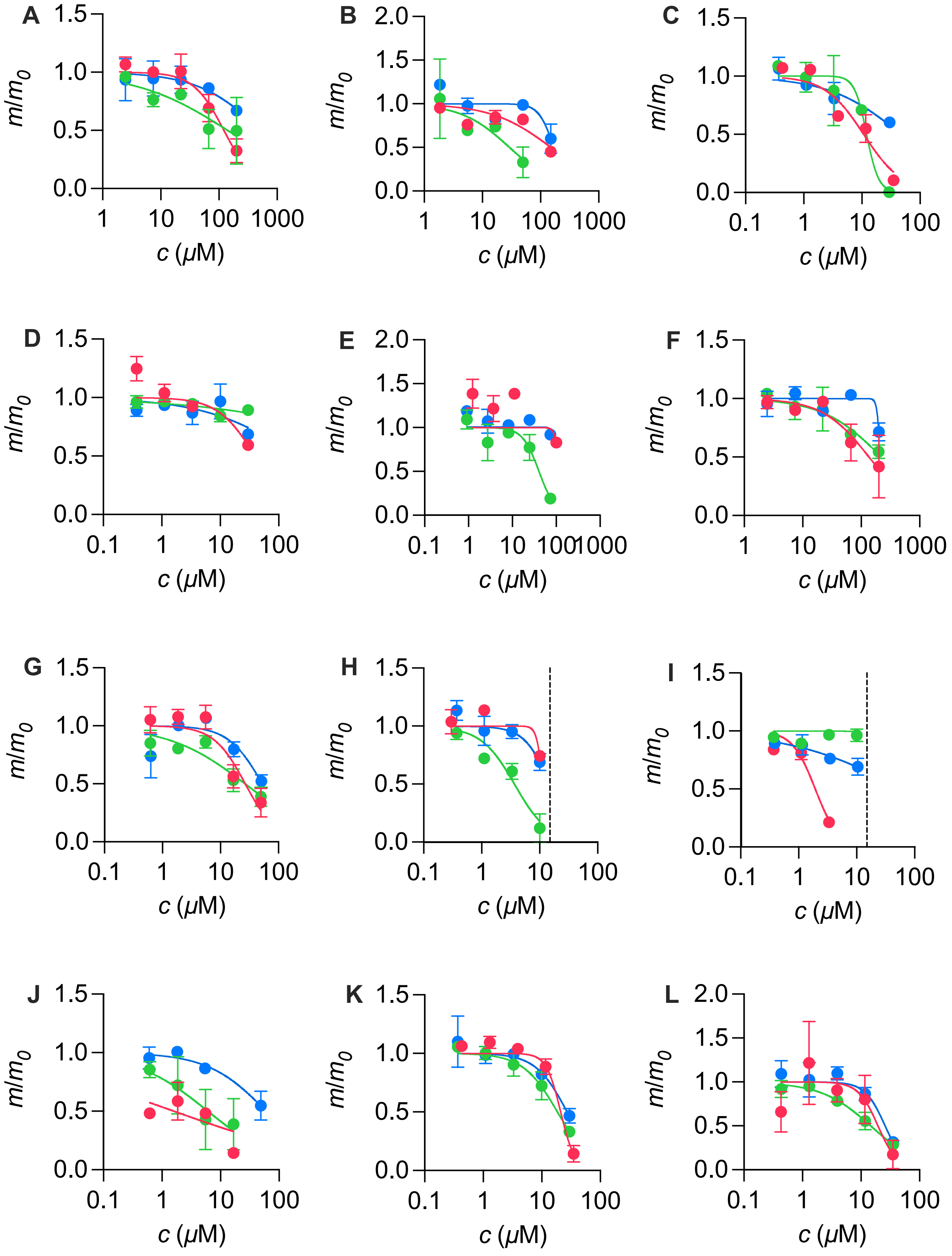

A
B
C
D
E
F
G
H
I
J
K
L
m/m0
c (μM)

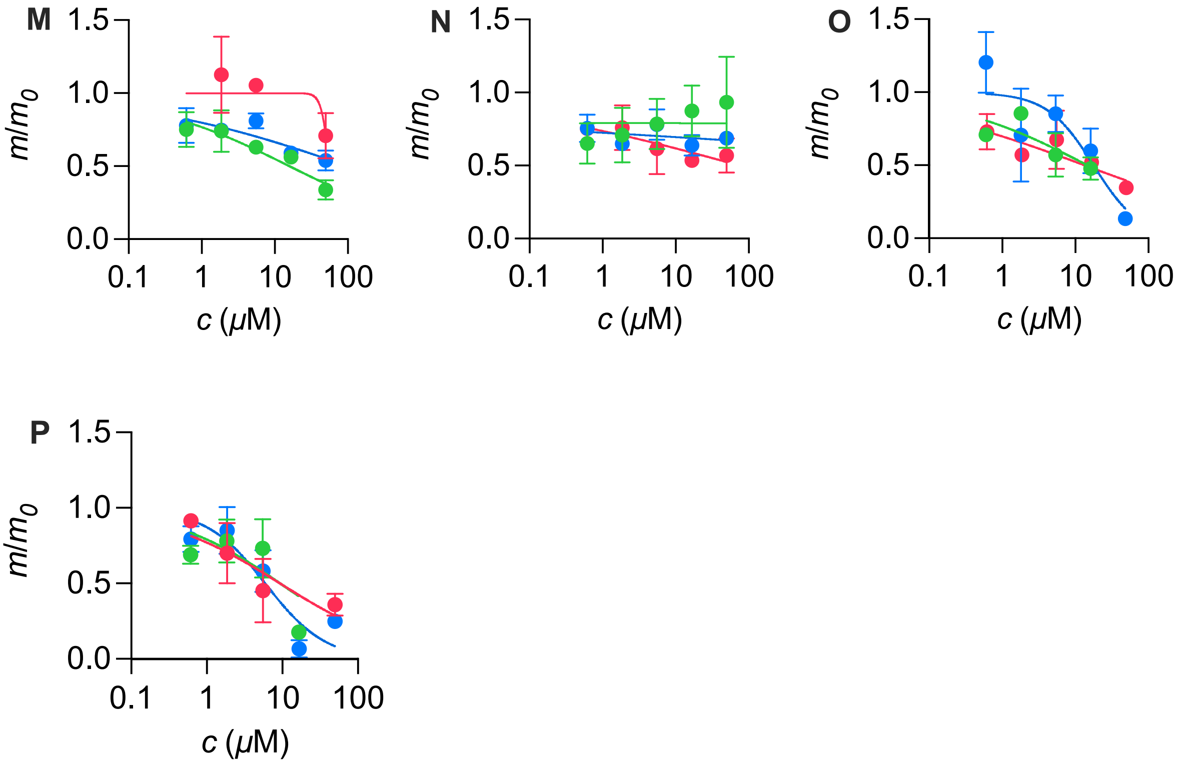

Figure S24. AHCHT data showing relative motility m/m_0 ± SEM of HK on collagen I (red), fibronectin (blue) and vitronectin (green) coated plates after 2 h of incubation as a function of the concentration of A) **1**, B) **2**, C) **3**, D) **4**, E) **5**, F) **6**, G) **7**, H) **8**, I) **9**, J) **10**, K) **11**, L) **12**, M) **13**, N) **14**, O) **15** and P) **16**. Dashed lines (c = 15 μM) represent the concentration at which the onset of toxicity was observed.

Table S6. Inhibition of HK cell motility on collagen I.[a]

Entry	I[b]	MIC (µM)[c]	IC_{50} (µM)[d]	*n*[e]
1	**1**	40	120 ± 30	1.6 ± 0.6
2	**2**	20	150 ± 120	0.8 ± 0.6
3	**3**	3	10 ± 3	1.3 ± 0.5
4	**4**	11	>30	-
5	**5**	100	>100	-
6	**6**	30	140 ± 50	1.0 ± 0.5
7	**7**	10	30 ± 5	1.6 ± 0.7
8	**8**	9	>10	-
9	**9**	<0.4	2.0 ± 0.6	1.0 ± 0.7
10	**10**	<0.6	2.0 ± 0.5	0.3 ± 0.1
11	**11**	10	20 ± 5	4 ± 1
12	**12**	10	20 ± 8	3 ± 2
13	**13**	45	>50	-
14	**14**	<0.4	>50	-
15	**15**	<0.4	15 ± 10	0.3 ± 0.2
16	**16**	<0.4	10 ± 5	0.5 ± 0.2

[a]Data obtained after 2 h of incubation. [b]Inhibitors. [c]Concentration needed to reach 15% inhibition. [d]Concentration needed to reach 50% inhibition (best fit ± SEM). [e]Hill coefficient for inhibition of cellular motility (best fit ± SEM). Data corresponding to the dose response curves presented in Figure S24.

7.2. Fibronectin

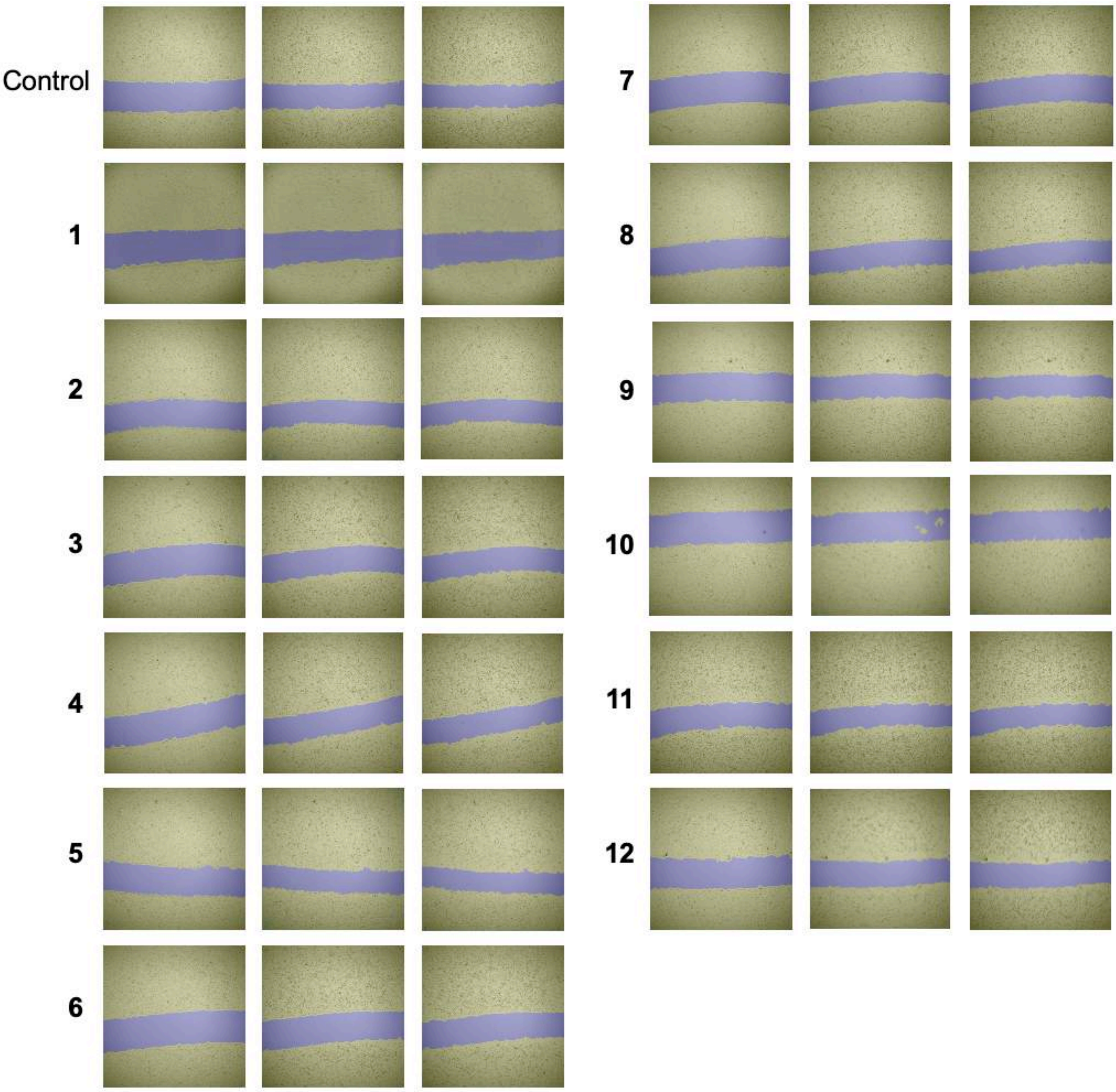

Figure S25. AHCHT images with overlayed masks of HK cells on fibronectin taken at 1 h (left), 8 h (middle) and 14 h (right) in the presence of **1** (200 µM), **2** (150 µM), **3** (30 µM), **4** (30 µM), **5** (75 µM), **6** (200 µM), **7** (50 µM), **8** (3 µM), **9** (3 µM), **10** (50 µM), **11** (30 µM) and **12** (35 µM). Scale bar: 1 mm.

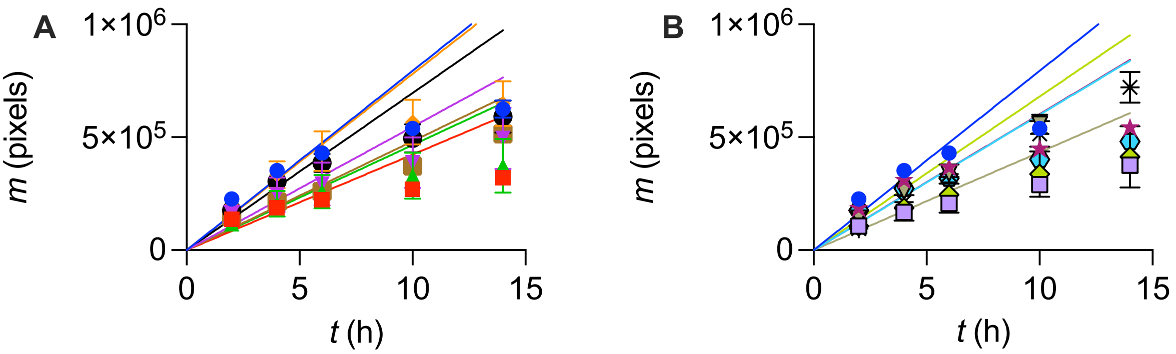

Figure S26. Motility as a function of time for HK cells on fibronectin. (A) Control (dark blue circles), **1** (red squares, 200 µM), **2** (green triangles, 150 µM), **3** (purple inverted triangles, 30 µM), **4** (orange diamonds, 30 µM), **5** (black circles, 75 µM), **6** (brown squares, 200 µM). (B) **7** (purple squares, 50 µM), **8** (bordeaux stars, 3 µM), **9** (olive inverted triangles, 3 µM), **10** (lime diamonds, 50 µM), **11** (black asterisks, 30 µM) and **12** (light blue hexagons, 35 µM).

Table S7. Inhibition of HK cell motility on fibronectin.[a]

Entry	I[b]	MIC (μM)[c]	IC_{50} (μM)[d]	*n*[e]
1	**1**	60	>200	-
2	**2**	100	>150	-
3	**3**	5	>30	-
4	**4**	10	>30	-
5	**5**	-	-	-
6	**6**	70	>200	-
7	**7**	15	50 ± 15	2 ± 1
8	**8**	10	>10	-
9	**9**	2	>10	-
10	**10**	10	>50	-
11	**11**	10	30 ± 7	1.6 ± 0.7
12	**12**	15	25 ± 5	3 ± 1
13	**13**	<0.6	>50	-
14	**14**	-	-	-
15	**15**	5	20 ± 5	1.4 ± 0.6
16	**16**	1	6 ± 2	1.1 ± 0.3

[a]Data obtained after 2 h of incubation. [b]Inhibitors. [c]Concentration needed to reach 15% inhibition. [d]Concentration needed to reach 50% inhibition (best fit ± SEM). [e]Hill coefficient for inhibition of cellular motility (best fit ± SEM). Data corresponding to the dose response curves presented in Figure S24.

7.3. Vitronectin

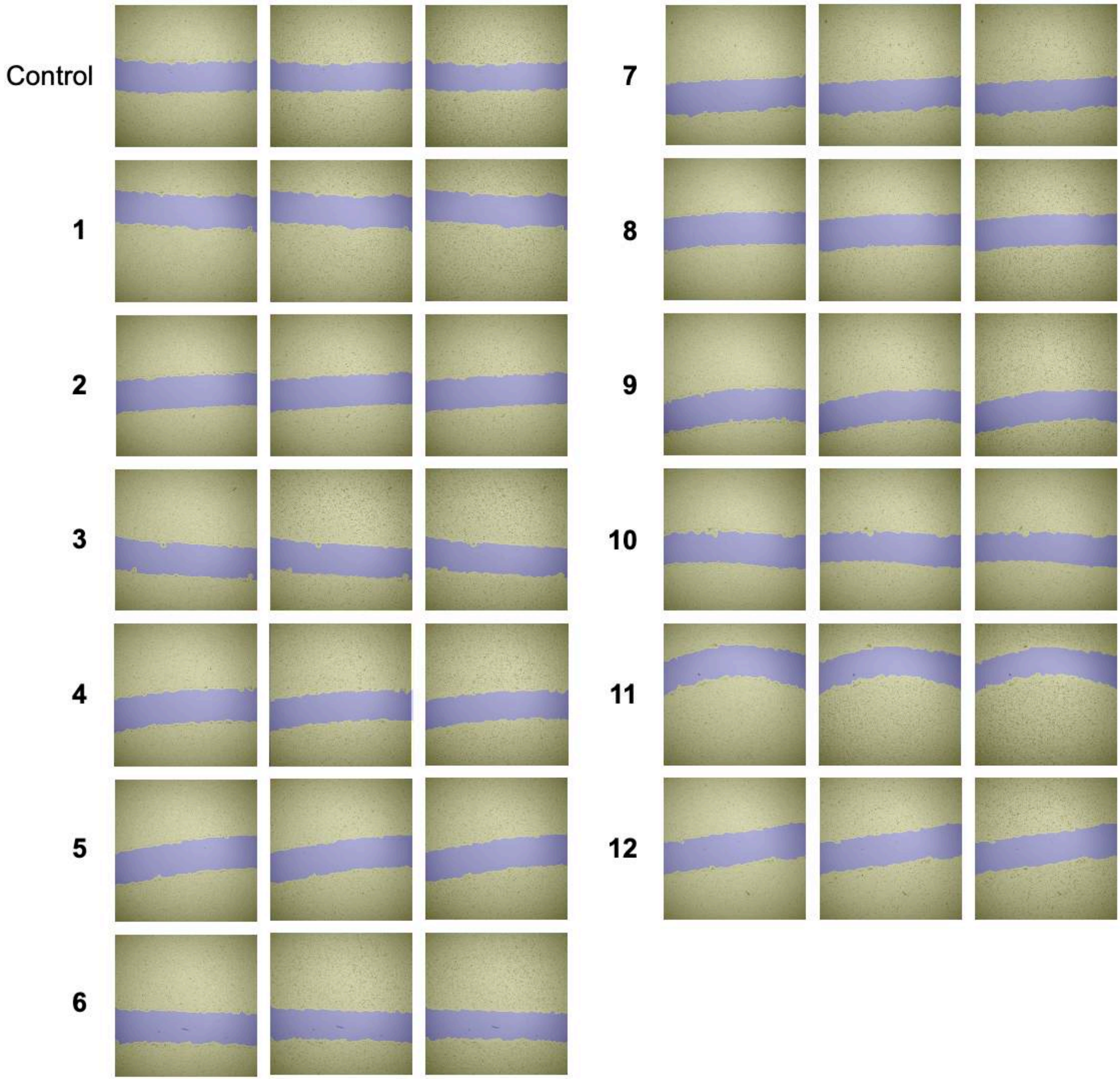

Figure S27. AHCHT images with overlayed masks of HK cells on vitronectin taken at 1 h (left), 8 h (middle) and 14 h (right) in the presence of **1** (200 µM), **2** (150 µM), **3** (30 µM), **4** (30 µM), **5** (75 µM), **6** (200 µM), **7** (50 µM), **8** (3 µM), **9** (3 µM), **10** (17 µM), **11** (30 µM) and **12** (35 µM). Scale bar: 1 mm.

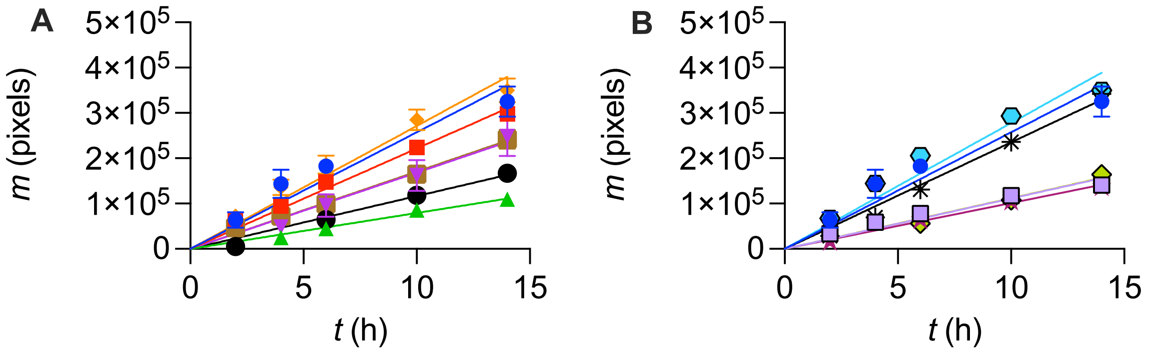

Figure S28. Motility as a function of time for HK cells on vitronectin. (A) Control (dark blue circles), **1** (red squares, 200 μM), **2** (green triangles, 150 μM), **3** (purple inverted triangles, 35 μM), **4** (orange diamonds, 30 μM), **5** (black circles, 75 μM), **6** (brown squares, 200 μM). (B) Control (dark blue circles), **7** (purple squares, 50 μM), **8** (bordeaux stars, 3 μM), **9** (olive inverted triangles, 3 μM), **10** (lime diamonds, 17 μM), **11** (black asterisks, 30 μM) and **12** (light blue hexagons, 35 μM).

Table S8. Inhibition of HK cell motility on vitronectin.[a]

Entry	I[b]	MIC (μM)[c]	IC_{50} (μM)[d]	n[e]
1	**1**	10	140 ± 60	0.5 ± 0.3
2	**2**	10	30 ± 15	1.0 ± 0.7
3	**3**	5	10 ± 4	4 ± 2
4	**4**	-	-	-
5	**5**	20	40 ± 10	2 ± 1
6	**6**	30	>200	-
7	**7**	2	30 ±15	0.7 ± 0.3
8	**8**	1	5 ± 1	2 ± 1
9	**9**	-	-	-
10	**10**	<0.6	6 ± 4	0.7 ± 0.4
11	**11**	5	20 ± 6	2 ± 1
12	**12**	5	15 ± 5	1.0 ± 0.4
13	**13**	<0.6	15 ± 5	1.2 ± 0.3
14	**14**	-	-	-
15	**15**	<0.6	20 ± 10	0.4 ± 0.4
16	**16**	1	10 ± 5	0.6 ± 0.5

[a]Data obtained after 2 h of incubation. [b]Inhibitors. [c]Concentration needed to reach 15% inhibition. [d]Concentration needed to reach 50% inhibition (best fit ± SEM). [e]Hill coefficient for inhibition of cellular motility (best fit ± SEM). Data corresponding to the dose response curves presented in Figure S24.

8. Inhibition of Cell Motility in the Presence of Serum

Inhibition of cell motility was tested in the presence of 2.5% FBS on collagen I in all cell lines. The motility inhibition was measured according to the procedure described in 4.2. and analyzed as described in section 4.3. The masks obtained for each cell lines are shown in Figures S29 (MDA-MB-231), S32 (MCF-7) and S35 (HeLa Kyoto). Application of Equation (S1) afforded the motility which was plotted at a fixed concentration overtime as shown in Figures S30 (MDA-MB-231), S33 (MCF-7) and S36 (HeLa Kyoto). The relative motility (m/m_0) at different concentrations was calculated to plot dose-response curves (Figures S31 (MDA-MB-231), S34 (MCF-7) and S37 (HeLa Kyoto)). The fit of these curves to Equation (S2) afforded parameters such as the concentration needed to reach 50% inhibition (IC_{50}), the concentration needed to reach 15% inhibition (MIC) and the Hill coefficient (n) for inhibition of cellular motility (Tables S9 (MDA-MB-231), S10 (MCF-7) and S11 (HeLa Kyoto)). A heatmap comparing three cell lines is displayed in Figure S38.

8.1. MDA-MB-231 Cells

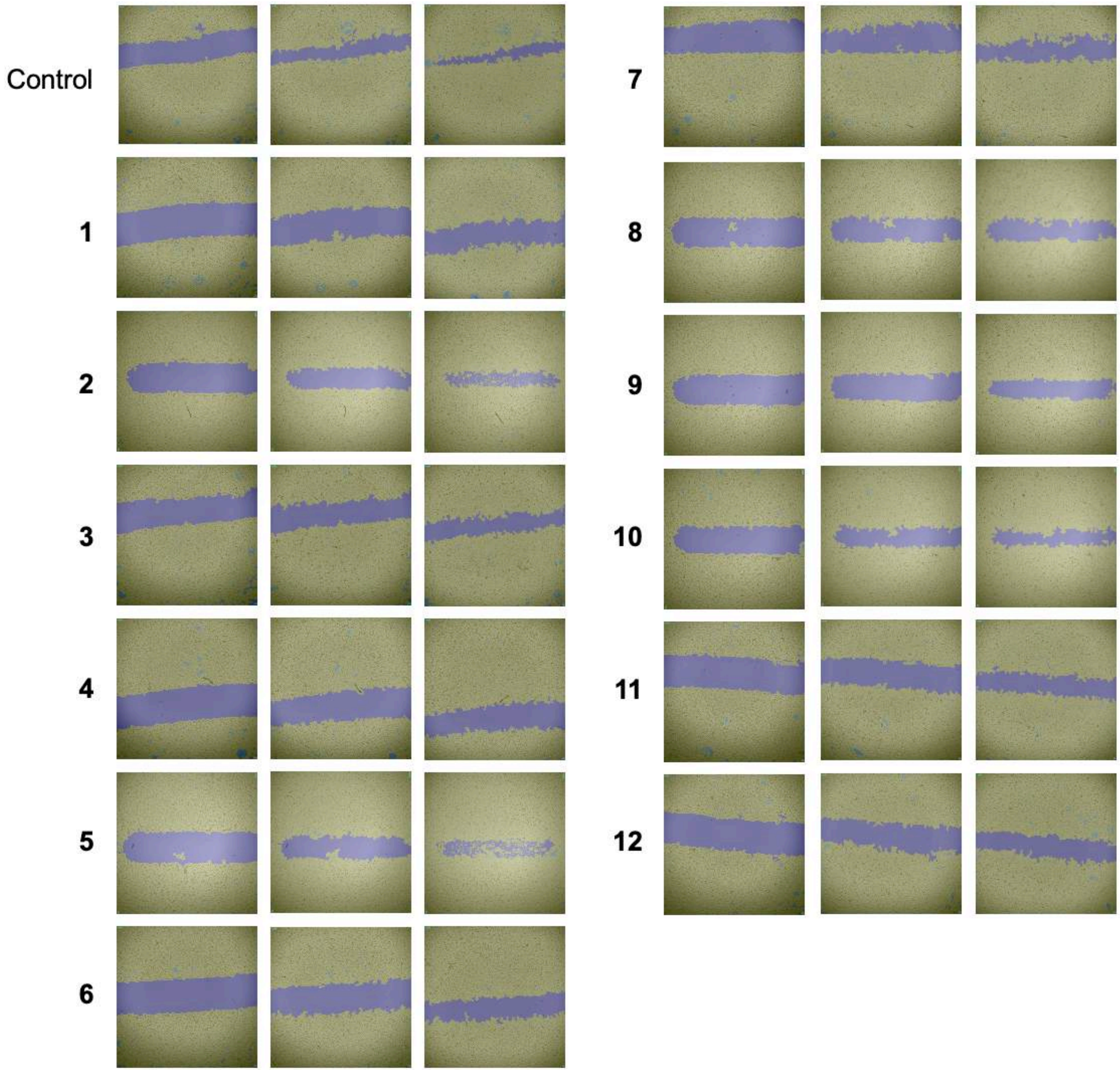

Figure S29. AHCHT images with overlayed masks of MDA-MB-231 cells on collagen I in 2.5% FBS taken at 1 h (left), 8 h (middle) and 14 h (right) in the presence of **1** (200 µM), **2** (150 µM), **3** (17 µM), **4** (17 µM), **5** (100 µM), **6** (200 µM), **7** (50 µM), **8** (3 µM), **9** (3 µM), **10** (50 µM), **11** (30 µM) and **12** (10 µM). Scale bar: 1 mm.

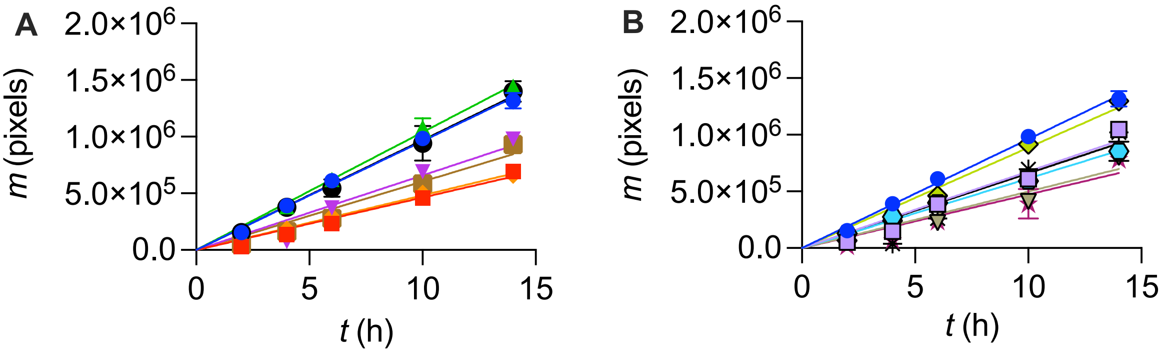

Figure S30. Motility as a function of time for MDA-MB-231 cells on collagen I in 2.5% FBS. (A) Control (dark blue circles), **1** (red squares, 200 μM), **2** (green triangles, 150 μM), **3** (purple inverted triangles, 17 μM), **4** (orange diamonds, 17 μM), **5** (black circles, 100 μM), **6** (brown squares, 200 μM). (B) **7** (purple squares, 50 μM), **8** (bordeaux stars, 3 μM), **9** (olive inverted triangles, 3 μM), **10** (lime diamonds, 50 μM), **11** (black asterisks, 30 μM) and **12** (light blue hexagons, 10 μM).

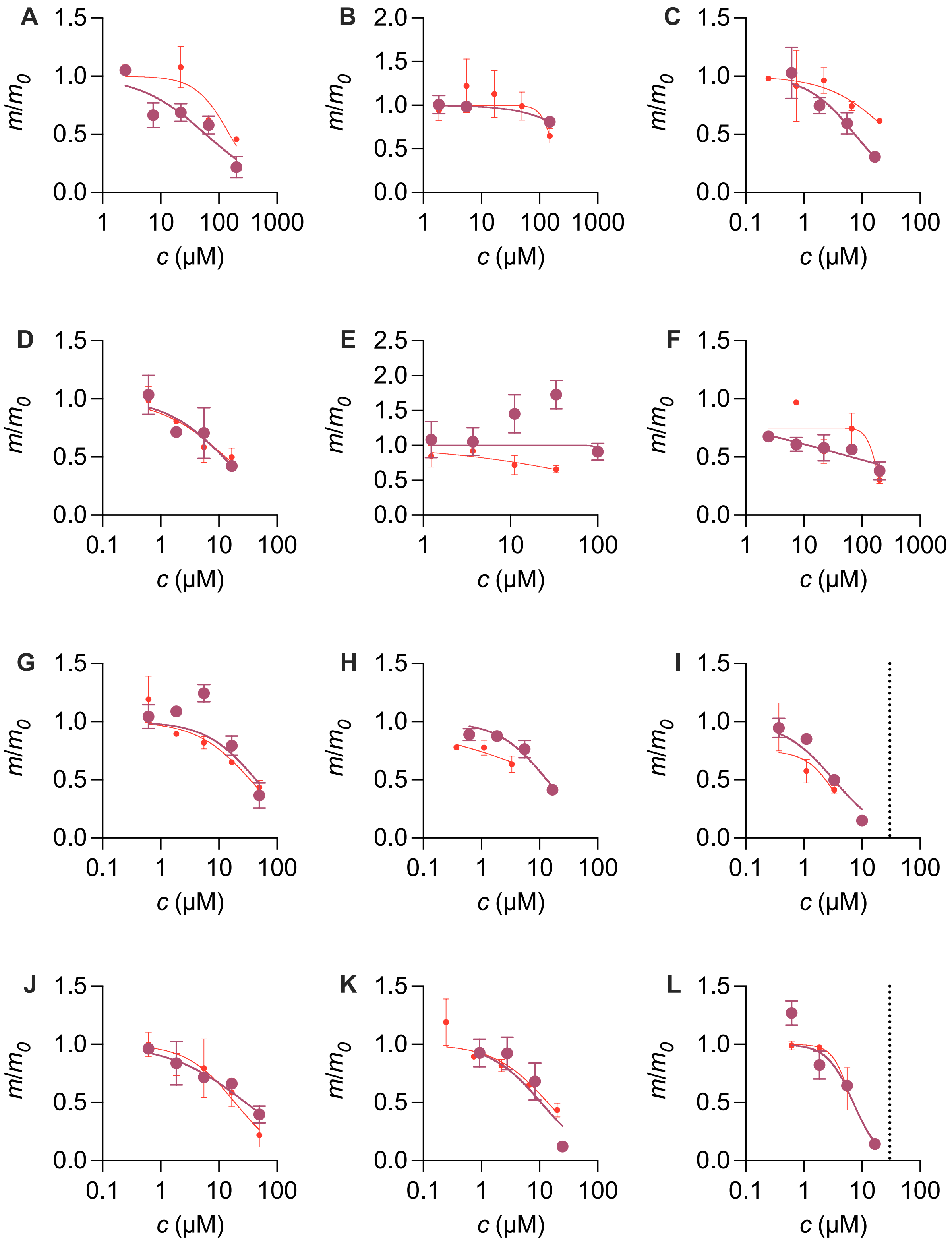

Figure S31. AHCHT data showing relative motility m/m_0 ± SEM of MDA-MB-231 cells on collagen I in 0% FBS (red circles) and 2.5% FBS (dark red circles, bold) after 2 h of incubation as a function

of the concentration of A) **1**, B) **2**, C) **3**, D) **4**, E) **5**, F) **6**, G) **7**, H) **8**, I) **9**, J) **10**, K) **11**, L) **12**. Dotted lines (c = 30 μM) represent the concentration at which the onset of toxicity was observed.

Table S9. Inhibition of cell motility of MDA-MB-231 on collagen I in 2.5% serum.[a]

Entry	I[b]	MIC (μM)[c]	IC_{50} (μM)[d]	n[e]
1	**1**	6	60 ± 20	0.8 ± 0.2
2	**2**	110	>150	0.9 ± 0.3
3	**3**	1	10 ± 2	1.0 ± 0.4
4	**4**	2	10 ± 5	1.0 ± 0.4
5	**5**	-	>100	-
6	**6**	<3	70 ± 50	0.2 ± 0.1
7	**7**	8	40 ± 20	1.0 ± 0.5
8	**8**	1	15 ± 4	1.0 ± 0.3
9	**9**	<0.4	3 ± 1	1.0 ± 0.4
10	**10**	2	30 ± 15	0.7 ± 0.3
11	**11**	2	10 ± 4	1.0 ± 0.6
12	**12**	3	8 ± 2	0.9 ± 0.4

[a]Data obtained after 2 h of incubation. [b]Inhibitors. [c]Concentration needed to reach 15% inhibition. [d]Concentration needed to reach 50% inhibition (best fit ± SEM). [e]Hill coefficient for inhibition of cellular motility (best fit ± SEM). Data corresponding to the dose response curves presented in Figure S31.

8.2. MCF-7 Cells

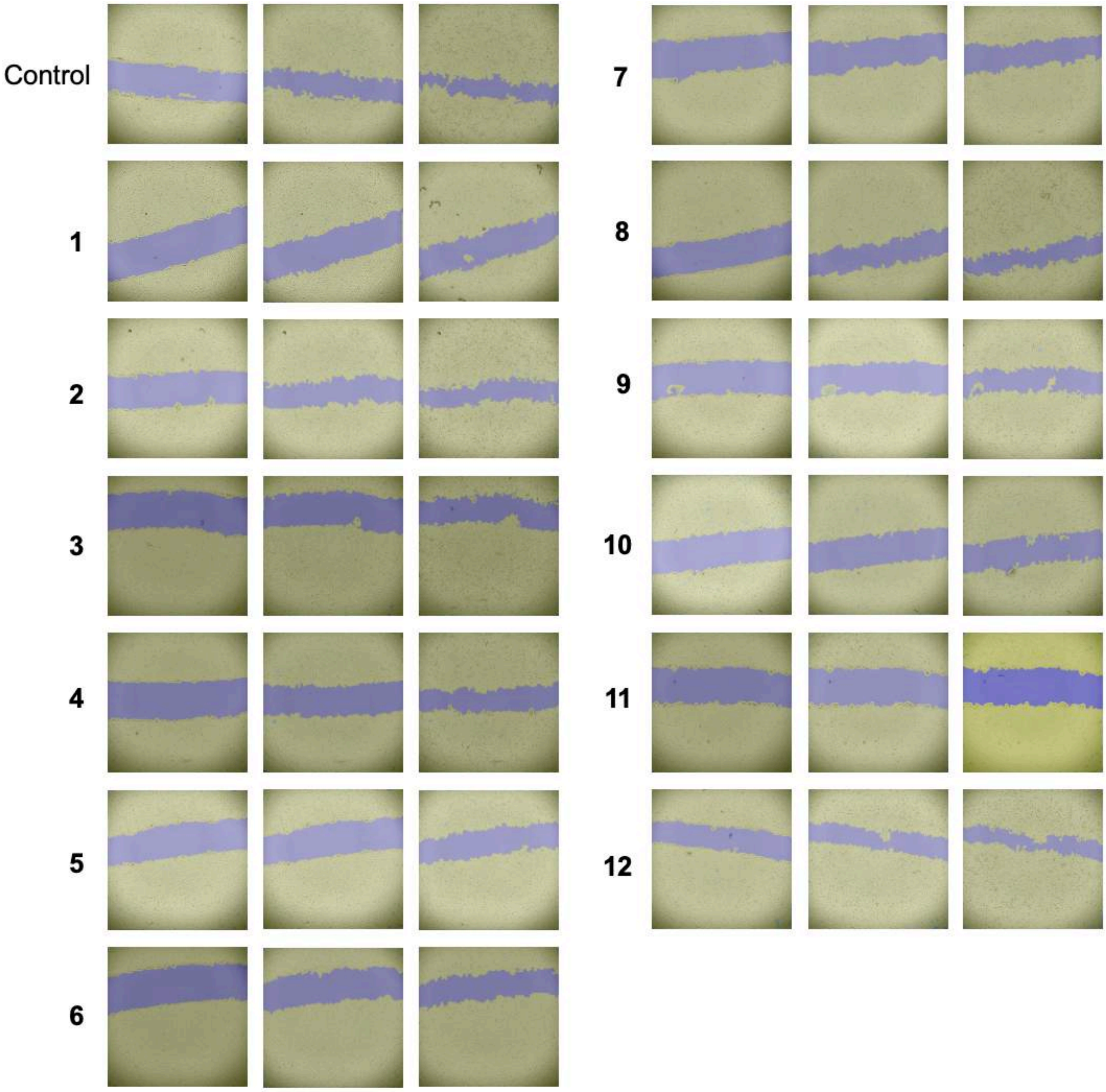

Figure S32. AHCHT images with overlayed masks of MCF-7 cells on collagen I in 2.5% FBS taken at 1 h (left), 8 h (middle) and 16 h (right) in the presence of **1** (200 µM), **2** (150 µM), **3** (10µM), **4** (5 µM), **5** (100 µM), **6** (200 µM), **7** (17 µM), **8** (3 µM), **9** (3 µM), **10** (17 µM), **11** (30 µM) and **12** (10 µM). Scale bar: 1 mm.

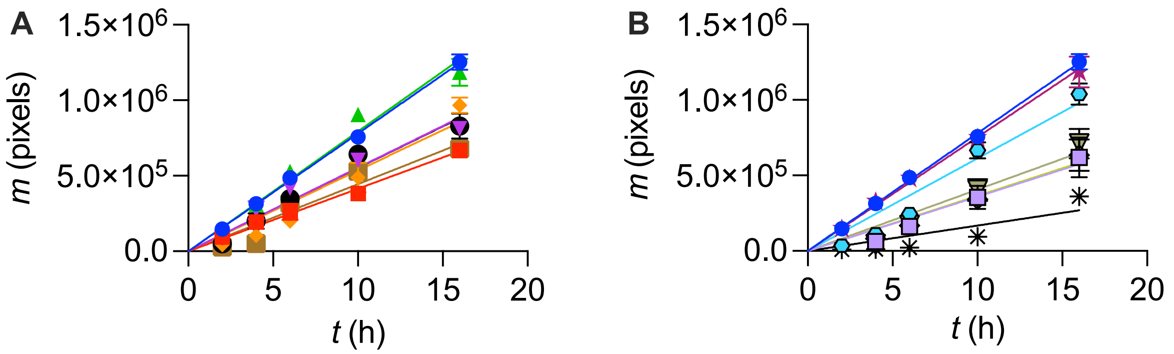

Figure S33. Motility as a function of time for MCF-7 cells on collagen I in 2.5% FBS. (A) Control (dark blue circles), **1** (red squares, 200 μM), **2** (green triangles, 150 μM), **3** (purple inverted triangles, 10 μM), **4** (orange diamonds, 5 μM), **5** (black circles, 100 μM), **6** (brown squares, 200 μM). (B) Control (dark blue, circles), **7** (purple squares, 17 μM), **8** (bordeaux stars, 3 μM), **9** (olive inverted triangles, 3 μM), **10** (lime diamonds, 17 μM), **11** (black asterisks, 30 μM) and **12** (light blue hexagons, 10 μM).

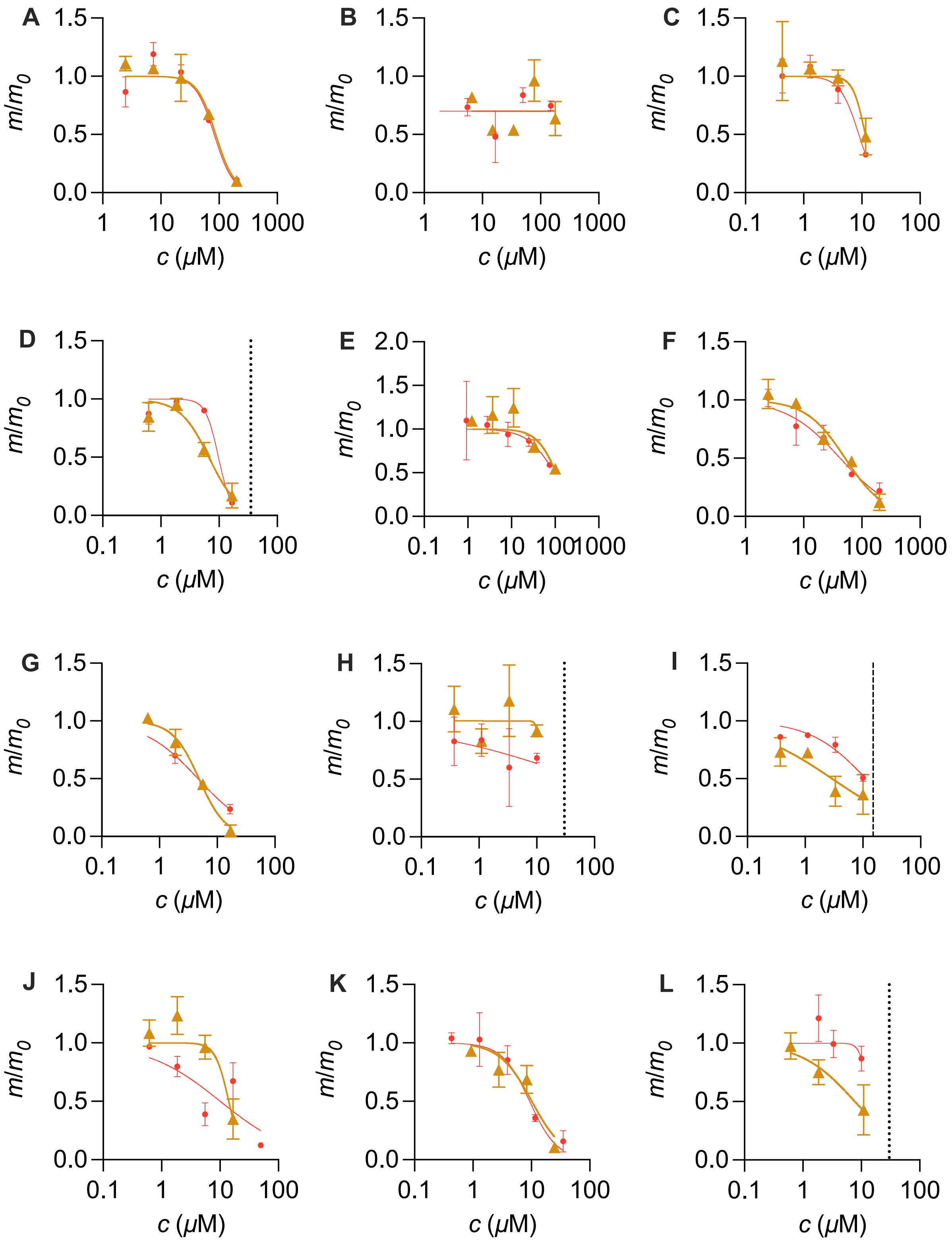

Figure S34. AHCHT data showing relative motility m/m_0 ± SEM of MCF-7 cells on collagen I in 0% FBS (red circles) and 2.5% FBS (brown triangles, bold) plates after 2 h of incubation as a function of

the concentration of A) **1**, B) **2**, C) **3**, D) **4**, E) **5**, F) **6**, G) **7**, H) **8**, I) **9**, J) **10**, K) **11**, L) **12**. Dashed (c = 15 μM) and dotted lines (c = 30 μM) represent the concentration at which toxicity was observed.

Table S10. Inhibition of cell motility of MCF-7 on collagen I with 2.5% serum.[a]

Entry	I[b]	MIC (μM)[c]	IC_{50} (μM)[d]	n[e]
1	**1**	50	90 ± 50	1.0 ± 0.4
2	**2**	-	-	-
3	**3**	7	10 ± 1	1.0 ± 0.9
4	**4**	3	7 ± 1	2.0 ± 0.4
5	**5**	40	100 ± 30	2 ± 1
6	**6**	10	50 ± 8	1.1 ± 0.2
7	**7**	2	5 ± 1	2.0 ±0.4
8	**8**	-	-	-
9	**9**	<0.4	3 ± 1	0.6 ± 0.2
10	**10**	10	15 ± 10	1.0 ± 0.6
11	**11**	3	10 ± 2	2.0 ± 0.3
12	**12**	1	8 ± 3	1.0 ± 0.3

[a]Data obtained after 2 h of incubation. [b]Inhibitors. [c]Concentration needed to reach 15% inhibition. [d]Concentration needed to reach 50% inhibition (best fit ± SEM). [e]Hill coefficient for inhibition of cellular motility (best fit ± SEM). Data corresponding to the dose response curves presented in Figure S34.

8.3. HeLa Kyoto Cells

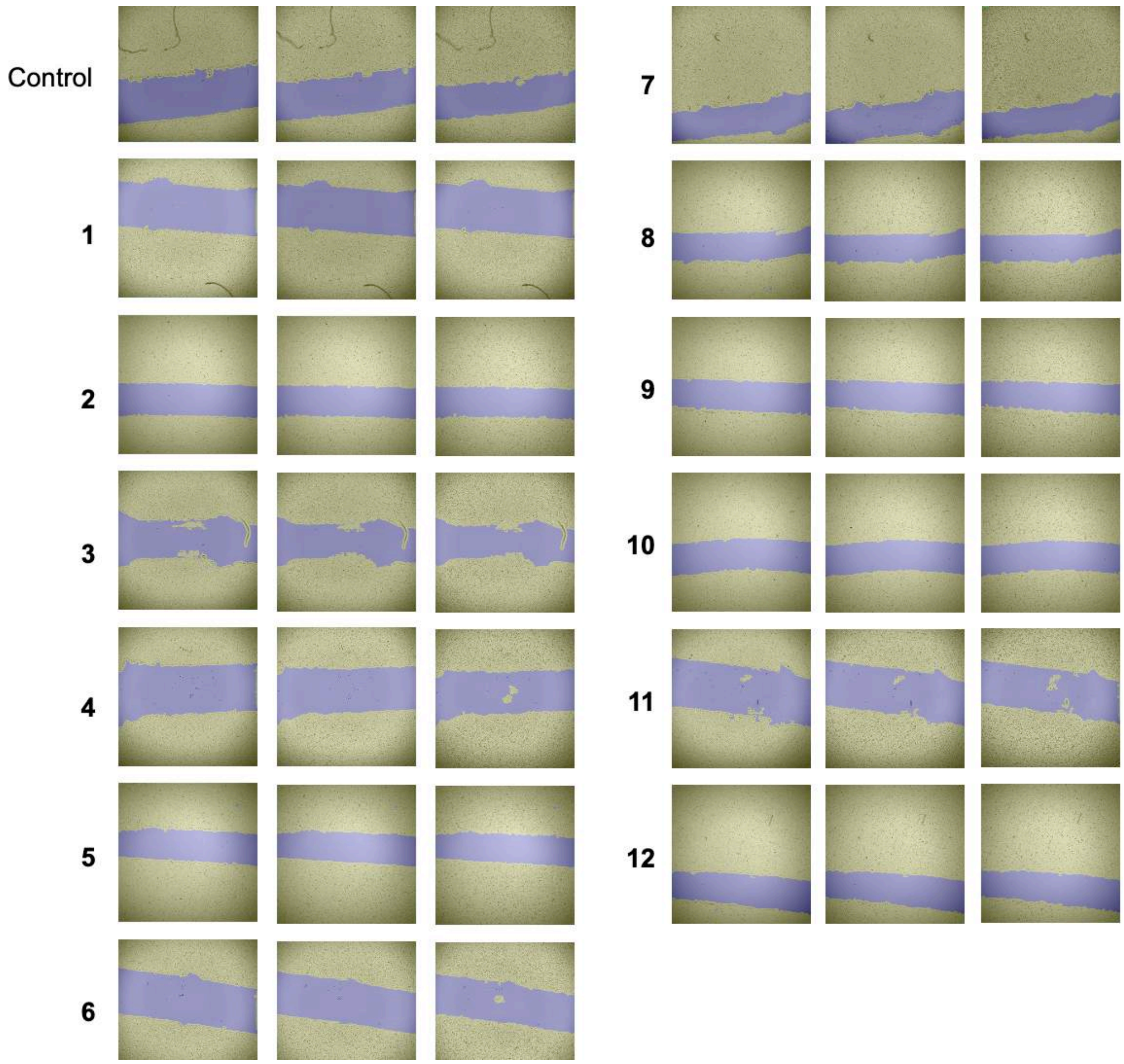

Figure S35. AHCHT images with overlayed masks of HeLa cells on collagen I in 2.5% FBS taken at 1 h (left), 8 h (middle) and 14 h (right) in the presence of **1** (200 µM), **2** (150 µM), **3** (17 µM), **4** (30 µM), **5** (100 µM), **6** (200 µM), **7** (30 µM), **8** (3 µM), **9** (3 µM), **10** (17 µM), **11** (20 µM) and **12** (17 µM). Scale bar: 1 mm.

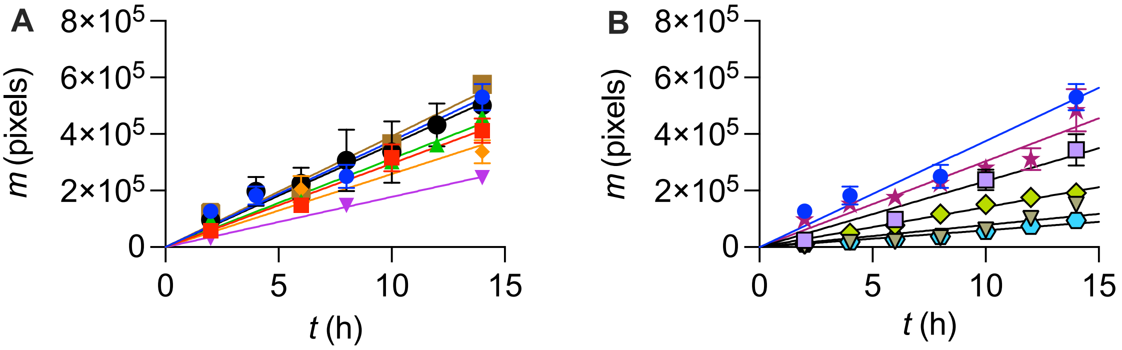

Figure S36. Motility as a function of time for HeLa cells on collagen I in 2.5% FBS. (A) Control (dark blue circles), **1** (red squares, 200 μM), **2** (green triangles, 150 μM), **3** (purple inverted triangles, 17 μM), **4** (orange diamonds, 30 μM), **5** (black circles, 100 μM), **6** (brown squares, 200 μM). (B) Control (dark blue, circles), **7** (purple squares, 30 μM), **8** (bordeaux stars, 3 μM), **9** (olive inverted triangles, 3 μM), **10** (lime diamonds, 17 μM), **11** (black asterisks, 20 μM) and **12** (light blue hexagons, 17 μM).

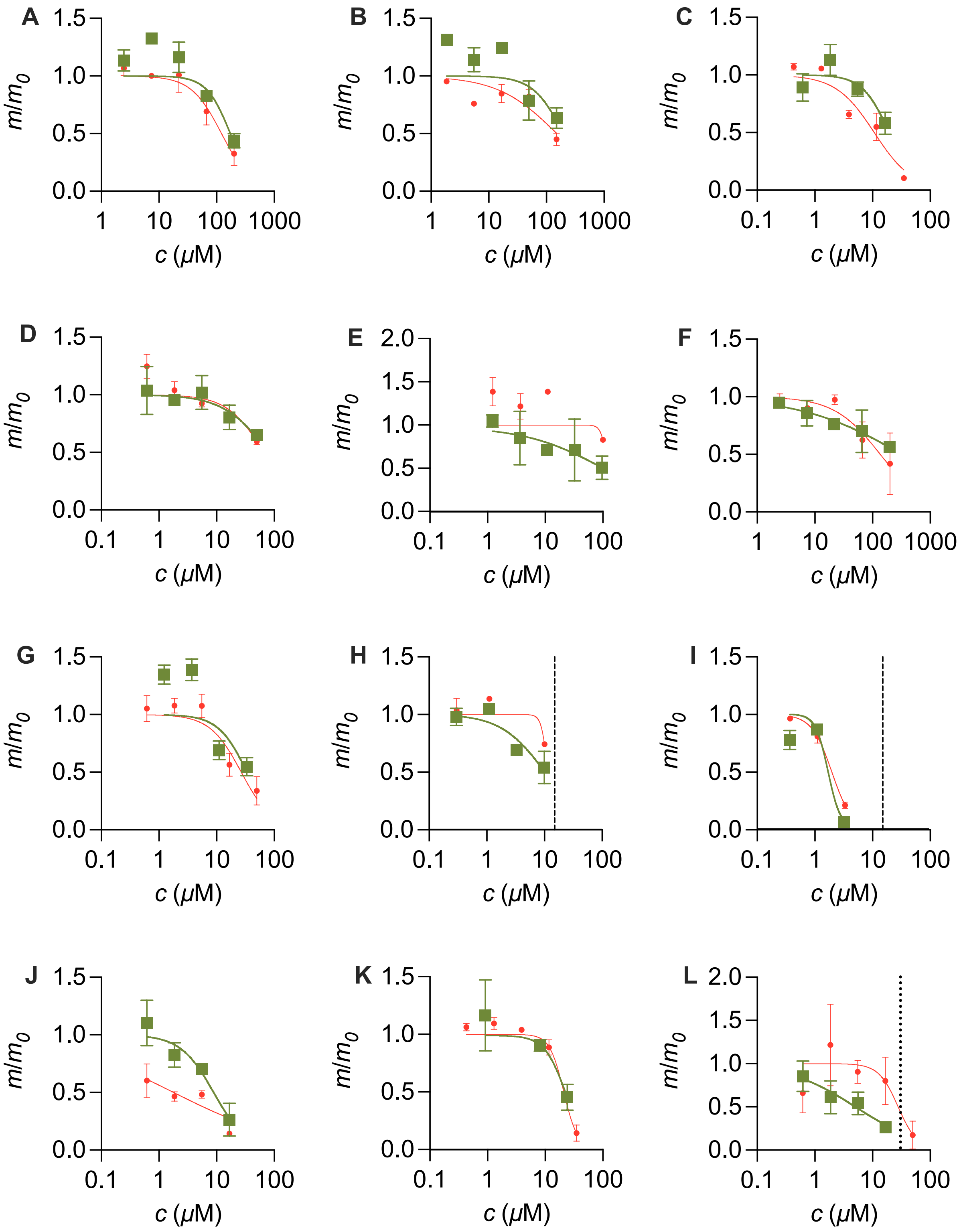

Figure S37. AHCHT data showing relative motility m/m_0 ± SEM of HeLa Kyoto cells on collagen I in 0% FBS (red circles) and 2.5% FBS (green squares, bold) plates after 2 h of incubation as a function of the concentration of A) **1**, B) **2**, C) **3**, D) **4**, E) **5**, F) **6**, G) **7**, H) **8**, I) **9**, J) **10**, K) **11**, L) **12**. Dashed

(c = 15 µM) and dotted lines (c = 30 µM) represent the concentration at which the onset of toxicity was observed.

Table S11. Inhibition of cell motility of HeLa Kyoto cells on collagen I with 2.5% serum.[a]

Entry	I[b]	MIC (µM)[c]	IC_{50} (µM)[d]	n[e]
1	**1**	75	170 ± 40	1.0 ± 0.6
2	**2**	70	>150	2 ± 1
3	**3**	8	20 ± 6	2 ± 1
4	**4**	15	>50	1.1 ± 0.6
5	**5**	10	>100	0.6 ± 0.4
6	**6**	10	>200	0.5 ± 0.2
7	**7**	10	30 ± 10	2 ± 1
8	**8**	2	10 ± 5	0.5 ± 0.4
9	**9**	<0.4	1.2 ± 0.7	1.0 ± 0.6
10	**10**	3	10 ± 3	1.0 ± 0.5
11	**11**	10	25 ± 5	2 ± 0.2
12	**12**	<0.6	5 ± 3	0.7 ± 0.3

[a]Data obtained after 2 h of incubation. [b]Inhibitors. [c]Concentration needed to reach 15% inhibition. [d]Concentration needed to reach 50% inhibition (best fit ± SEM). [e]Hill coefficient for inhibition of cellular motility (best fit ± SEM). Data corresponding to the dose response curves presented in Figure S37.

8.4. Heatmap

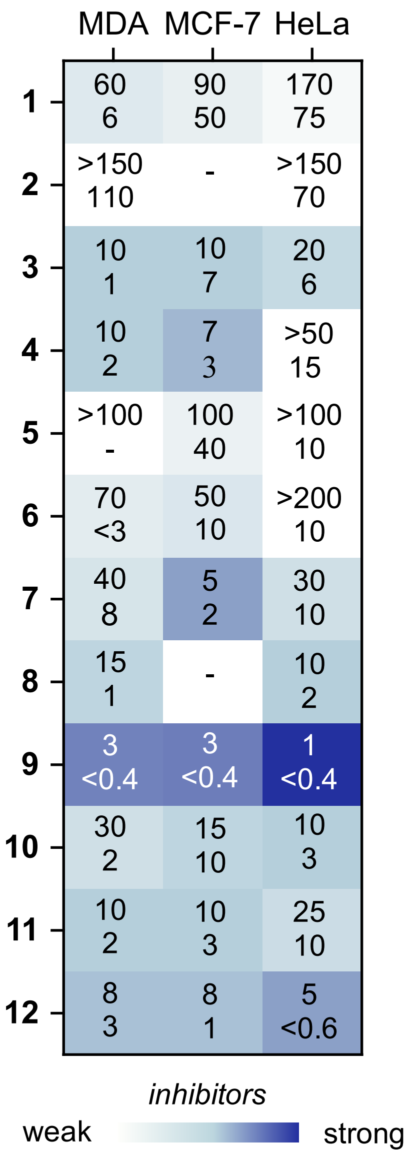

Figure S38. Heatmap for IC_{50} of candidates **1-12** to inhibit the motility of MDA-MB-231, MCF-7 and HK cells on collagen I (C) measured 2 hours after addition in DMEM medium containing 2.5% of fetal bovine serum (FBS; Tables S9-S11). Numbers show IC_{50} (top) and MIC (bottom), both in µM; -: inactive.

9. Single Cell Motility

9.1. General Procedure for Single Cell Motility Inhibition

MDA-MB-231 cells were seeded at 7 × 10^3 cells/well in DMEM + 10% FBS on coated µ-Plate 96-well Black ibiTreat sterile and kept at 37 °C under 5% CO_2 atmosphere overnight. Next day, the cells were washed with PBS (1 x 2 mL/well) and the medium was exchanged to DMEM (4 x 150 µL), using a plate washer (Biotek EL406®) while keeping a final volume of 135 µL/well. The inhibitor solution was prepared in FluoroBrite DMEM which was then added to the cells (15 µL/well) using an electronic multichannel pipette to reach a final volume of 150 µL/well, and the plate was imaged using an automated confocal microscope. For this experiment, a transmitted light (TL) image at the center of the well was acquired at 4x (3.5 x 3.5 mm) every 5 minutes for 6 h.

9.2. Data Analysis for Single Cell Motility Inhibition

Analysis of single cell motility was performed using the Track Objects tool of MetaXpress® software (Figure S39A). Each isolated cell was marked as an object with an area of 20 x 20 with a search region for the same object on next time point image of 50 x 50 (Figure S39B). Each cell track can be easily validated visually on the time point movie before export of the data. The data presented are the result of the analysis of 30 cells. Quadruplicates were performed for each condition.

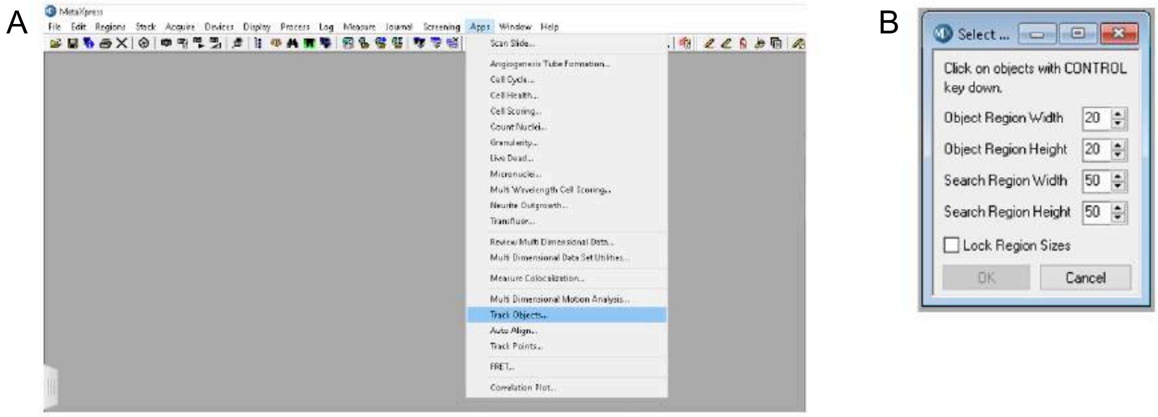

Figure S39. Software interface of MetaXpress with Track Objects tool highlighted (A) and object and search regions used in the tracking method (B).

10. Knockdown of ITGB1

10.1. General Procedure for the Knockdown of ITGB1 in HK Cells

For the knockdown of integrin β1, reverse transfection method was performed with a final concentration of 5 nM siRNA siPOOL based on the procedure of transfection provided by siTOOLs Biotech. Briefly, siRNA diluted in reduced serum medium Opti-MEM was pre-mixed with Lipofectamine RNAiMAX and Opti-MEM for 15 minutes at room temperature. The pre-mixed solution of siRNA was transferred to a μ-Plate 96-well Black ibiTreat sterile (30 μL/well). HeLa Kyoto cells were then seeded at 1800 cells/well and incubated at 37 °C under 5% CO_2 atmosphere for 2-3 days.

10.2. General Procedure for the Uptake in Knocked-down HK Cells

The cells were washed with PBS (3 x 3 mL/well) and then with FluoroBrite DMEM using a plate washer, keeping a final volume of 100 μL/well. The corresponding reporter **17** (10 mM, DMSO) was diluted in FluoroBrite DMEM and added (50 μL) to the cells keeping a final concentration from 5 μM and a final volume of 150 μL/well (DMSO < 0.01%). The cells were incubated for 1 hour at 37 °C under 5% CO_2 atmosphere. The cells were washed in the same manner described in 10.1 to remove the excess of the fluorescent reporter. After the addition of the nuclear stain Hoechst 33342 (50 μL, 30 μg/mL), the cells were incubated for 5 minutes at 37 °C under 5% CO_2 atmosphere. The cells were washed for the last time with PBS (9 x 3 mL/well) and kept in PBS for imaging. The fluorescence intensity was captured on a IXM-C automated microscope acquiring 4 images/well using a 10× objective lens with 2 channels, blue for Hoechst 33342 (377/50 nm excitation filter; 477/60 nm emission filter) and green for FITC transporter (475/34 nm excitation filter; 536/40 nm emission filter).

10.3. General Procedure for Integrin Knockdown Quantification by Immunofluorescence

After the imaging of transporters (10.2.), PBS was manually removed from the wells and the cells were fixed with a solution of 5% PFA for 15 minutes at room temperature. The cells were

washed with PBS (9 x 3 mL/well) using the plate washer and treated for 1 hour with mouse monoclonal anti-integrin β1 antibody in PBS containing 1% BSA and 0.05% saponin (70 µL per well, 1/200 antibody). The cells were washed with PBS (9 × 3 mL/well) using the plate washer and treated with a solution of the secondary antibody with a fluorescent dye in PBS (80 µL per well, 1/400; Alexa Fluor® 647 AffiniPure Donkey Anti-Mouse IgG (H+L)). The cells were washed one last time with PBS (9 × 3 mL/well) and then imaged on a IXM-C automated microscope acquiring 9 images per well using a 20× objective lens with 2 channels, blue for Hoechst 33342 (377/50 nm excitation filter; 477/60 nm emission filter) and red for the secondary antibody (620/50 nm excitation filter; 690/50 nm emission filter).

11. Pattern Generation

Presumably, TMU is a multi-target process as concluded in previous works (refs. S1, S2, S4 and S8) as well as in this work. These results suggest a mechanism with a complex network of proteins operating in a dynamic fashion. To elaborate on possible cooperativity patterns involved in inhibition, MICs (Tables S1-S8) and switching half-windows (C_R) were subjected to pattern generation (Figures S40 and S41). Hill coefficients were considered less suitable for this purpose.

Switching half-windows (C_R) are an adaptation of a cooperativity parameter described in ref. S9, which measures the logarithmic ratio between the concentration needed to reach 90% of binding and the concentration needed to reach 10% of binding. This parameter allows to identify positive (small C_R) or negative (large C_R) cooperative mechanisms of substrate-protein binding. Here, the IC_{50} (Tables S1-S8) and MIC (Tables S1-S8, Figure S40) were used to calculate switching half-windows C_R using Equation (S3).

$$C_R = \log_{10}(\frac{IC_{50}}{MIC}) \quad \text{(S3)}$$

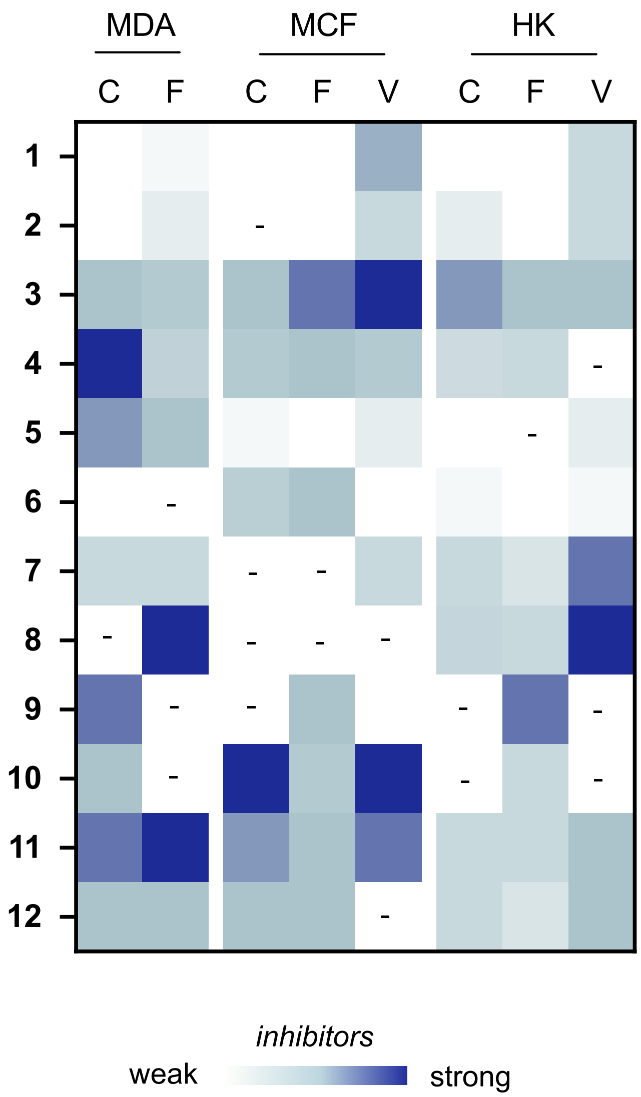

Figure S40. Heatmap for the MIC of candidates **1-12** to inhibit the motility of MDA-MB-231, MCF-7 and HK cells on collagen I (C), fibronectin (F) and vitronectin (V), measured 2 hours after addition (Tables S1-S8).

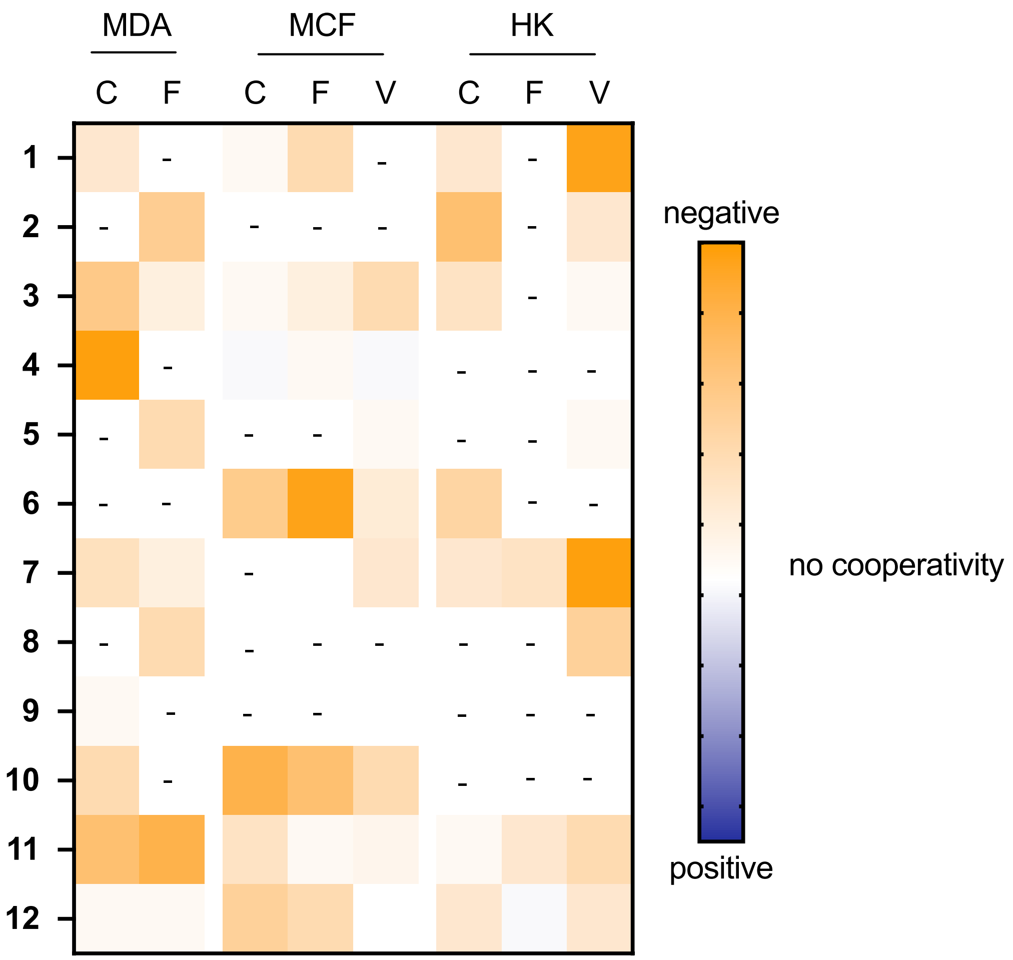

Figure S41. Heatmap for the switching half-window (C_R) of candidates **1-12** to inhibit the motility of MDA-MB-231, MCF-7 and HK cells on collagen I (C), fibronectin (F) and vitronectin (V), measured 2 hours after addition (Tables S1-S8, Equation (S3)). Here $C_R = 0.25$ was arbitrarily taken as non cooperative. Small switching half-windows (blue) were of interest because they can indicate positive cooperativity, large switching windows (orange) were particularly noteworthy because they can, besides negative cooperativity and competing effects (solubility, etc), indicate the presence of several active sites involved in exchange cascades.[S9]

12. Supporting References

(S1) Shybeka, I.; Maynard, J. R. J.; Saidjalolov, S.; Moreau, D.; Sakai, N.; Matile, S. Dynamic Covalent Michael Acceptors to Penetrate Cells: Thiol-Mediated Uptake with Tetrel-Centered Exchange Cascades, Assisted by Halogen-Bonding Switches. *Angew. Chem. Int. Ed.* **2022**, e202213433.

(S2) Cheng, Y.; Pham, A.-T.; Kato, T.; Lim, B.; Moreau, D.; López-Andarias, J.; Zong, L.; Sakai, N.; Matile, S. Inhibitors of Thiol-Mediated Uptake. *Chem. Sci.* **2021**, *12*, 626–631.

(S3) Jin, W. B.; Xu, C.; Cheng, Q.; Qi, X. L.; Gao, W.; Zheng, Z.; Chan, E. W. C.; Leung, Y.-C.; Chan, T. H.; Wong, K.-Y.; Chen, S.; Chan, K.-F. Investigation of Synergistic Antimicrobial Effects of the Drug Combinations of Meropenem and 1,2- Benzisoselenazol-3(2H)-One Derivatives on Carbapenem-Resistant Enterobacteriaceae Producing NDM-1. *Eur. J. Med. Chem.* **2018**, *155*, 285–302.

(S4) Lim, B.; Kato, T.; Besnard, C.; Poblador Bahamonde, A. I.; Sakai, N.; Matile, S. Pnictogen-Centered Cascade Exchangers for Thiol-Mediated Uptake: As(III), Sb(III) and Bi(III) Expanded Cyclic Disulfides as Inhibitors of Cytosolic Delivery and Viral Entry. *JACS Au* **2022**, *2*, 1105-1114.

(S5) Gu, J.; Xiao, B-X.; Chen, Y-R.; Li, Q-Z.; Ouyang, Q.; Du, W.; Chen, Y-C. Interrupted Morita-Baylis-Hillman-Type Reaction of α-Substituted Activated Olefins. Org. Lett. **2018**, *20*, 2088–2091.

(S6) Frei, R.; Wodrich, M. D.; Hari, D. P.; Borin, P.-A.; Chauvier, C.; Waser, J. Fast and Highly Chemoselective Alkynylation of Thiols with Hypervalent Iodine Reagents Enabled through a Low Energy Barrier Concerted Mechanism. *J. Am. Chem. Soc.* **2014**, *136*, 16563–16573.

(S7) Zong, L.; Bartolami, E.; Abegg, D.; Adibekian, A.; Sakai, N.; Matile, S. Epidithiodiketopiperazines: Strain-Promoted Thiol-Mediated Cellular Uptake at the Highest Tension. *ACS Cent. Sci.* **2017**, *3*, 449–453.

(S8) Abegg, D.; Gasparini, G.; Hoch, D. G.; Shuster, A.; Bartolami, E.; Matile, S.; Adiberian, A. Strained Cyclic Disulfides Enable Cellular Uptake by Reacting with the Transferin Receptor. *J. Am. Chem. Soc.* **2017**, *139*, 231-238.

(S9) Hunter, C. A.; Anderson, H. L. What is Cooperativity? *Angew. Chem. Int. Ed.* **2009**, *48*, 7488-7499.

The original data that support the findings of this study are openly available:

https://doi.org/10.5281/zenodo.7783830
